# *AmMADS47* of *Agropyron mongolicum* negatively regulates drought tolerance in rice

**DOI:** 10.3389/fpls.2025.1514134

**Published:** 2025-05-01

**Authors:** Bobo Fan, Hushuai Nie, Xiaolei Li, Yu Ma, Ersuo Lv, Jing Wu, Xiuxiu Yan, Yongqing Zhai, Yan Zhao, Jie Liu, Xiaohong Du, Yanhong Ma

**Affiliations:** ^1^ Agricultural College, Inner Mongolia Agricultural University, Hohhot, China; ^2^ Ecological Environment College, Baotou Teachers’ College, Baotou, China; ^3^ Inner Mongolia Academy of Agricultural & Animal Husbandry Sciences, Hohhot, China; ^4^ College of Grassland, Resources and Environment, Inner Mongolia Agricultural University, Hohhot, China

**Keywords:** *Agropyron mongolicum*, drought stress, *AmMADS47*, reactive oxygen species, transcriptional regulation

## Abstract

MADS-box transcription factors are important regulators of plant abiotic stress response. Despite the exceptional drought tolerance of *Agropyron mongolicum*, research on the MADS-box transcription factors governing simulate drought stress in this species are limited. In this study, overexpressing *AmMADS47* in rice resulted in reduced drought tolerance. Transcriptome sequencing of wild-type (WT) and transgenic rice (OE) at 0 hours of drought and wild-type (WTD) and transgenic rice (OED) at 24 hours of osmotic stress revealed 21,521 differentially expressed genes (DEGs) totally. Further analysis of the top 20 enriched pathways of the DEGs between OE and WT, and between OED and WTD showed that phenylpropanoid biosynthesis and glutathione metabolism were the shared pathways most enriched in DEGs, and photosynthesis-antenna proteins were the shared pathway with the highest enrichment score and significance. Gene regulation in response to osmotic stress was analyzed in the three pathways, showing that, compared to WTD, OED exhibited up-regulation of a few drought-sensitive genes, while most genes positively regulating drought in WTD were down-regulated in OED. Collectively, these results highlight the crucial role of *AmMADS47* in modulating the synthesis of key enzymes and the expression patterns of drought-responsive genes in three candidate pathways in rice, ultimately reducing drought resistance in rice.

## 1 Introduction

*Agropyron mongolicum*, a resilient Gramineous perennial grass thriving in deserts, is mainly distributed in Russia, Mongolia, and northern China, and it has a high nutritional value with a protein content of 18.64% (Du et al., 2017). Its stem leaves are soft with good palatability for animal husbandry. Evolving the over years, it has developed a high ecological value because of its drought resistance, cold resistance, and suitability for dry sandy areas (Zhao et al., 2010; Zhang et al., 2019a). It plays an important role in ecological restoration, wind prevention, and sand fixation, serving as a vital genetic resource for enhancing stress tolerance in Gramineae crops (Zhang et al., 2019b; Fan et al., 2022). Climate change exerts profound impacts on agricultural systems, with global warming exacerbating the frequency of extreme climatic events such as droughts while simultaneously driving the progressive expansion of arid regions. Research indicates that drought-induced production losses approximate 7% in affected agricultural areas (Huang et al., 2015; Lesk et al., 2016). Therefore, in-depth investigation of wild drought-tolerant genetic resources holds critical significance for agricultural adaptation strategies. The exacerbation of global warming and environmental drought stress poses challenges to realizing the full genetic potential of crops (Boyer, 1982; Esfahanian et al., 2017). Drought stress induces alterations in plant phenotype, physiology, and molecular level, activating drought response genes (Chandra et al., 2021). Under drought stress, plants express and activate drought response genes, initiating a series of signal transduction and metabolic pathways that shape plant phenotypic and physiological changes. Recent research has revealed that transcription factors, such as WRKY, NAC, ERF, and DREB, are critical to drought tolerance (Chandra et al., 2021). However, research on key MADS-box transcription factors and their regulatory mechanisms in *A. mongolicum* under simulate drought stress is scarce.

MADS-box transcription factors play important regulatory roles in plant growth, development, and responses to environmental adversity (Natalia et al., 2019; Okay et al., 2024; Mirzaghaderi, 2024). These factors can be categorized into two types: Type I, featuring SRF-like structural domain, and Type II, comprising the MEF2-like structural domain (highly conserved), the I structural domain (containing 35 amino acids, lowly conserved), the K-box (containing 65–70 amino acids, moderately conserved), and the C structural domain (lowly conserved) (Wei et al., 2015; Luis et al., 1996; Becker et al., 2003). *OsMADS26*, identified as a regulator in stress responses like drought and pathogen infection, exhibited up-regulation in rice induced by mannitol. However, overexpression in rice or *Arabidopsis* resulted in severe adversity phenotypes, including slow root and branch growth, sterility, and pale green coloration, reducing drought tolerance in rice (Lee et al., 2008a; Khong et al., 2015). *OsMADS23* and *OsMADS25* genes in rice, induced by mannitol, may involve in regulating drought stress (Puig et al., 2013). Overexpression of *OsMADS23* remarkably enhanced, but knockout of the gene greatly reduced the drought and salt tolerance in rice plants. Further, *OsMADS23* was shown to promote the biosynthesis of endogenous ABA and proline by activating the transcription of target genes *OsNCED2, OsNCED3, OsNCED4* and *OsP5CR* that are key components for ABA and proline biosynthesis (Li et al., 2021). Through comparative genome-wide analysis of MADS-box transcription factor families in rice, Abdullah-Zawaw found that the abiotic and biotic stress-responsive cis-regulatory element type and distribution patterns in the promoter regions of rice MADS-box, and showed the important role of MADs-box in rice stress responsiveness (Abdullah-Zawawi et al., 2021). miR444 is target to a MADS-box transcription factor *OsMADS27* in rice, transgenic rice plants expressing miR444 target mimic improved rice root growth, but overexpression of miRNA-resistant *OsMADS27* improved root development and tolerance to abiotic stresses, while its silencing suppressed root growth (Pachamuthu et al., 2022). *PgMADS41* and *PgMADS44* are involved in regulating root growth and development in *Panax* (Jiao et al., 2023). Overexpression of *SLMBP22* gene in tomatoes increased drought resistance, leading to changes in chlorophyll content, soluble sugar, starch and the transcript levels of genes related to chlorophyll development and metabolism, as revealed by real-time quantitative polymerase chain reaction (qRT-PCR) (Li et al., 2020). In *Sorghum bicolor*, the expression of MADS-box gene (*PTSb00221.1* and *PTSb00208.1*) in roots under water stress was up-regulated 5–10 fold (Aglawe et al., 2012). *DgMADS114* and *DgMADS115* genes from *Dactylis glomerata* improved stress tolerance when heterologously expressed in *Arabidopsis thaliana*, resulting in longer root length or higher survival rates under PEG and ABA (Yang et al., 2022). Research reports on the regulation of drought tolerance by the *MADS47* gene in plants remain limited. Related studies have only been identified in rice, where research demonstrates that the *OsMADS47* gene is predominantly expressed in plant tissues such as mature leaves, coleoptiles, and root elongation zones and promotes leaf sheath elongation while increasing leaf inclination angles. The *OsMADS47* gene acts as a negative regulator of BR signaling. Research shows that BRs can activate plant reactive oxygen species and phenylpropanoid metabolism (Ryo et al., 2022; Han et al., 2022; Fornara et al., 2008). All of the above studies have shown that MADS-box plays an important role in regulating stress in plants, however, there is little mining and application of MADS-box genes in the strongly drought-tolerant *A. mongolicum*. It is important to understand the MADS-box genes that regulate drought in *A. mongolicum*, a highly drought-tolerant crop.

In our preliminary study, we identified the MADS-box transcription factor (*AmMADS47*) as a key player in regulating simulate drought stress in *A. mongolicum* (Fan et al., 2022). In this study, the function of the *AmMADS47* gene was analyzed by observing the phenotype of rice plant overexpressing *AmMADS47*, determining its expression levels in roots, stems, and leaves, and evaluating ROS accumulation. Furthermore, the effects of *AmMADS47* overexpression on downstream regulated genes and candidate metabolic pathways in rice were analyzed through transcriptome sequencing. This study contributes to further understanding of the regulatory mechanisms of Poaceae grass MADS-box transcription factors under abiotic stresses and provides new insights for improving stress tolerance in Poaceae grasses and crops.

## 2 Materials and methods

### 2.1 Basic bioinformatics prediction of the *AmMADS47*

Mature seeds of *A. mongolicum* were collected from the Inner Mongolia Agricultural University’s (Hohhot, China) forage test station in the Inner Mongolia Autonomous Region. The amino acid sequence of the *AmMADS47* gene from *A. mongolicum*’s Coding sequence (CDS) was retrieved using the ORF Finder tool on the NCBI website (https://www.ncbi.nlm.nih.gov/orffinder/). A comparative analysis was performed by using the CDS sequence on NCBI to find genes that are highly homologous to the *AmMADS47* gene (https://blast.ncbi.nlm.nih.gov/Blast.cgi). Furthermore, the structural domain of the *AmMADS47* protein were analyzed using CDD available on NCBI (https://www.ncbi.nlm.nih.gov/Structure/bwrpsb/bwrpsb.cgi?cdsid=QM3-qcdsearch-346AED479377E099&tdata=qopts). The prediction of phosphorylation sites was conducted using the NetPhos website (https://services.healthtech.dtu.dk/services/NetPhos-3.1/). To unravel the protein’s secondary and tertiary structures, analyses were carried out through the SOPMA (https://npsa-prabi.ibcp.fr/cgi-bin/npsa_automat.pl?page=/NPSA/npsa_sopma.html) and SWISS-MODEL online platforms (https://swissmodel.expasy.org/interactive#sequence). Known and predicted physical and functional protein-protein interactions were obtained from the Search Tool for the Retrieval of Interacting Genes/Proteins (STRING) database (http://string-db.org/) by “Single/Multiple Proteins by Sequence”.

### 2.2 Overexpression vector construction of *AmMADS47* gene

Total RNA from *A. mongolicum* leaves was extracted using TRNzol Universal Reagent (Tiangen biotech, DP424, Beijing, China). Subsequently, the concentration and purity of the RNA were detected using NanoDrop One, and the RNA was reverse transcribed into cDNA using the FastQuant RT Kit (Tiangen biotech, KT106, Beijing, China), serving as a cloning template. The cloning process utilized primers KL-F-*AmMADS47* and KL-R-*AmMADS47*, with the KL-R-*AmMADS47* removing the terminator (**Supplementary Table S1**). The amplification system and procedure followed the steps outlined in **Supplementary Table S2**. The purified *AmMADS47* target fragment was recovered using the TIANgel Midi Purification Kit (Tiangen biotech, DP209, Beijing, China). Subsequent ligation of the *AmMADS47* target fragment and the cloning vector was carried out following the guidelines of the pEASY-Blunt Simple Cloning Kit (Transgen, CB111, Beijing, China) (**Supplementary Figure S1A**). Positive clones were subjected to double verification using both universal primers (M13-F: GTAAAACGACGGCCAGT, M13-R: CAGGAAACAGCTATGAC) and specific primers (KL-F-*AmMADS47*, KL-R-*AmMADS47)*. The amplification band of the universal and specific primers were 776 bp and 675 bp, respectively.

The PBI121-EGFP vector plasmid (Miaoling biology, Wuhan, China) and *AmMADS47* target fragment were digested with *Xho I* and *Sal I* restriction enzymes (Thermo Fisher Scientific Inc., China) (**Supplementary Table 3**), and the resulting products underwent purification and recovery. The PBI121-EGFP was ligated with *AmMADS47* (**Supplementary Table S3**), and the ligated product was transformed into Top10 competent cells (Weidi, DL1010, Shanghai, China), adhering strictly to the provided instructions. The monoclonal bacterial solution was sent to BGI Genomics Co., Ltd. for sequencing, and the correctly sequenced recombinant plasmid was named PBI121-*AmMADS47*-EGFP. The plasmid was further extracted using the TIANprep Mini plasmid Kit (Tiangen biotech, DP103, Beijing, China) and then transformed into *Agrobacterium Tumefaciens* GV3101 strain (Weidi, AC1001, Shanghai, China). This vector was subsequently used for subcellular localization and genetic transformation.

### 2.3 Subcellular localization of PBI121-*AmMADS47*-EGFP

The seeds of *Nicotiana benthamiana* were kept in the laboratory. The subcellular localization experiment utilized *N. benthamiana* as the experimental material, cultured in an artificial climate chamber (MGC-450HP-2) under conditions of 25°C and a 14 h light cycle for one month. *Agrobacterium tumefaciens* containing PBI121-*AmMADS47*-EGFP and PBI121-EGFP were cultured until reaching an OD_600_ value of 0.6. The bacterial cultures were then collected (5,000 rpm, 5 min), and the clear liquid was discarded. The bacterial pellet was resuspended in an equal volume of special infection solution (Coolaber, SL0911, Beijing, China) for tobacco. Subsequently, acetosyringone (20 mg/L) was added, and the infection solution was left at room temperature for 30 min before being used for injecting tobacco leaves. The injected regions on the tobacco leaves were marked, and the infested tobacco plants were incubated in the dark for 24 hours, followed by a transfer to the MGC-450HP-2 chamber for an additional 48 hours. The lower epidermal sections of the tobacco leaves were then torn and observed under a fluorescence microscope (OLYMPUS BX53) to determine the subcellular localization of PBI121-*AmMADS47*-EGFP. Centrifugation was performed using a C1650-230V centrifuge (Beijing Lebote, China).

### 2.4 *Agrobacterium tumefaciens* mediates genetic transformation in rice

The seeds of Nipponbare (*Oryza. sativa L. ssp* japonica) were provided by Shaanxi Baiai Gene Information Technology Co., Ltd., China. The callus of Nipponbare was infected with *Agrobacterium tumefaciens* containing PBI121-*AmMADS47*-EGFP. The *Agrobacterium tumefaciens* were cultured in liquid LB medium containing kanamycin (50 mg/L) and rifampicin (20 mg/L) at 28°C with agitation at 200 rpm. A thermostatic oscillator (HZQ-Q, Henglong Instrument Co., Ltd.) was employed for constant temperature oscillation. The OD_600_ value of the bacterial solution was maintained at 0.5. After centrifugation at 5,000 rpm for 5 min, the supernatant was discarded, and the organisms were resuspended in an equal volume of 1/2 MS liquid medium. Acetosyringone (20 mg/L) was added to the suspension, and the mixture was left at room temperature for 30 min to facilitate subsequent genetic transformation of rice. The callus of rice was infected for 15 min, followed by co-culturing after removing excess bacterial liquid from the callus. Positive rice callus was screened, followed by differentiation and strengthening of seedlings. Positive identification indicated that PBI121-AmMADS47-EGFP was successfully constructed and transformed into *Agrobacterium tumefaciens* GV3101 (**Supplementary Figure S1B–D**). Overexpressed *AmMADS47* rice callus was obtained through two rounds of resistance callus screens (**Supplementary Figures S2A, B**). The seedlings resistant to callus differentiation were transferred to strong seedling mediums, leading to the development of transgenic rice lines (**Supplementary Figures S2C, D**). The formulation of the medium used in these experiments is shown in **Supplementary Table S4**. DNA of transgenic rice was extracted using the CTAB method, and positive rice was identified through polymerase chain reaction (PCR). DNA was extracted from rice leaves using the cetyltrimethylammonium bromide (CTAB) method to confirm positive rice lines, and the sequences of CaMV35s added CDS of *AmMADS47* was amplified. Positive rice plants were then transplanted into soil as seedlings and allowed to mature for seed harvesting.

### 2.5 Cultivation and treatment of rice

50 seeds each from the wild type and positive rice lines were selected. The seeds underwent a series of surface sterilization steps, involving a 30 s∼1.5 min soak in 75% alcohol, followed pouring out the alcohol. Subsequently, the seeds underwent a 1 min soak in a 2.5% sodium hypochlorite solution, with the removal of the solution, the addition of a new 2.5% sodium hypochlorite solution, and at 150 rpm, a 20 min wash. The seeds were then transfer to a super clean table after removal of the sodium hypochlorite solution and underwent 15 rinses with sterilized ddH_2_O. The sterilized seeds were then germinated in the dark at 25 °C using filter paper in a germination box. After 5 days of seed germination, seedlings with uniform growth were selected and transplanted into nutritious soil with a 1 cm spacing between seedlings. The rice were cultured to the 4-leaf stage before being transferred to a hydroponic culture (nutrient solution: 2.37 g MS/L water). To induce simulate drought stress conditions, a 25% PEG-6,000 (W/V) treatment was administered, and samples were collected both before and 24 hours after the stress application. These samples were promptly placed in liquid nitrogen pre-cooled enzyme-free freezing tubes and then transferred to -80 °C for storage. Each sample type underwent three biological repeats to ensure robustness and reproducibility of the experimental results.

### 2.6 qRT-PCR in trans-*AmMADS47* rice

Total RNA was extracted from leaves of overexpressed rice subjected to both non-drought treatment and drought treatment for 24 hours (D24 h). The RNA concentration and purity were detected using NanoDrop One, followed by reverse transcription to cDNA using the FastQuant RT Kit (Tiangen biotech, KT106, Beijing, China). For qRT-PCR, upstream and downstream primers for the *AmMADS47* gene were F-*AmMADS47* “CAGGCTCGGATTACCACTCTTCAAC” and R-*AmMADS47* “TATTCAATGCAATGCGCGGGTTCAAC”. The *Actin* gene was used as an internal reference, with F-*Actin* “CAATGTGCCAGCTATGTATGTCGC” and R-*Actin* “TTCCCGTTCAGCAGTGGTAGTGAAG”. Primers were synthesized by Sangon Biotech Co., Ltd. (Shanghai, China). The qRT-PCR was performed with MonAmp SYBR Green qPCR Mix (Monad, MQ10201S, Wuhan, China) following strict protocol instructions. The relative expression levels were calculated using the 2^–ΔΔCT^ method (Livak and Schmittgen, 2001). Two rice lines with the highest expression were screened, and the expression levels of the *AmMADS47* gene in roots, stems, and leaves were determined by qRT-PCR, with three biological replicates established.

### 2.7 Characterization of drought resistance in trans-*AmMADS47* rice

After drought stress treatment, the plants whose leaves could grow upright, had fresh green color and showed no obvious wilting phenomenon were identified as “drought resistance” plants. For the assessment of oxidative stress in wild-type and overexpressed rice, leaves at the 4-leaf stage were subjected to both non-drought treatment and 25% PEG-6000 drought treatment for 24 hours. Nitrobule tetrazolium (NBT) and diaminobenzidine (DAB) staining were performed to observe O^2-^ and H_2_O_2_ accumulation in the leaves. NBT (SL18061) and DAB (SL1805) staining solutions were purchased from Beijing Coolaber Technology Co., Ltd., China. To study the effect of *AmMADS47* overexpression on rice root, rice lines exhibiting the highest expression of *AmMADS47* were selected, and root phenotype were observed. Parameters such as root length, root surface area, root volume, and root tip number of rice at the 4-leaf stage were statistically analyzed using the LA-S root analysis system (Wseen, Hangzhou, China). The malondialdehyde (MDA) assay kit (A003-1), proline (Pro) assay kit (A107-1-1), and catalase (CAT) assay kit (Visible light) were used to determine the physiological indexes of wild-type and OE-25 rice at the 4-leaf stage. These kits were purchased from Nanjing Jiancheng Bioengineering Institute, China. The test procedures strictly adhered to the instructions provided with each kit.

### 2.8 Transcriptome sequencing of overexpressed rice under drought stress

Total RNA was extracted from OE-25 (OE), WT, drought-treated 24-h OE-25 (OED), and WTD leaves using Trizol reagent (Invitrogen, CA, USA). The quantity and purity of total RNA in each sample were assessed using the RNA 1000 Nano LabChip Kit (Agilent, CA, USA). The integrity of total RNA was evaluated with the Agilent Bioanalyzer 2100 analyzer (RIN value > 7.0, OD value 260/280 ≥ 1.8). The qualified total RNA was enriched with Oligo (dT) magnetic beads to obtain poly (A) RNA, which was subsequently cut into small fragments by Fragmentation Buffer. The cut fragments were reverse transcribed into the final cDNA library using the mRNA Seq sample preparation kit (Illumina, San Diego, USA), with library quality undergoing rigorous control measures. Rice transcriptome sequencing was performed on the Illumina Novaseq 6000 platform. Raw data were processed using the FASTQ to obtain clean reads, and HISAT2 software was employed for mapping to the reference genome and calculation of gene expression (FPKM) (Kim et al., 2015; Chen et al., 2018). The read counts of each gene were obtained through HTSeq-count (Simon et al., 2015), and the gene was analyzed by PCA using *R* packet (v 3.2.0). Sequencing services were entrusted to Shanghai OE Biotech Co., Ltd.

### 2.9 Analysis of differentially expressed genes and pathways of overexpressed *AmMADS47*

DEGs were analyzed using DESeq2 (Love et al., 2014), and DEGs responsive to *AmMADS47* and osmotic stress were screened using criteria of *q* < 0.05, foldchange > 2, or foldchange < 0.5. Hierarchical clustering of DEGs was performed with *R* packet (v3.2.0) to analyze expression patterns of genes across different comparison groups. The expression profiles of the top 30 up-regulated and down-regulated genes were analyzed using ggrada. GO and KEGG pathway enrichment analysis of DEGs were used to screen terms and pathways responsive to *AmMADS47* while regulating osmotic stress. Pathway maps for the selected key pathways were generated, and an expression heat map was constructed for genes annotated to each pathway. The relative expression levels of genes within the candidate pathway were analyzed. The qRT-PCR kit and template were consistent with those described in section 2.6. Primer sequences are shown in **Supplementary Table S5**.

### 2.10 Statistical analysis

Each graphical represents the results from at least three repeats, and the values are displayed as the mean ± SD. Statistical significance was determined using Student’s t-test.

## 3 Results

### 3.1 *AmMADS47* gene sequence and subcellular localization analysis

**Figure 1:**
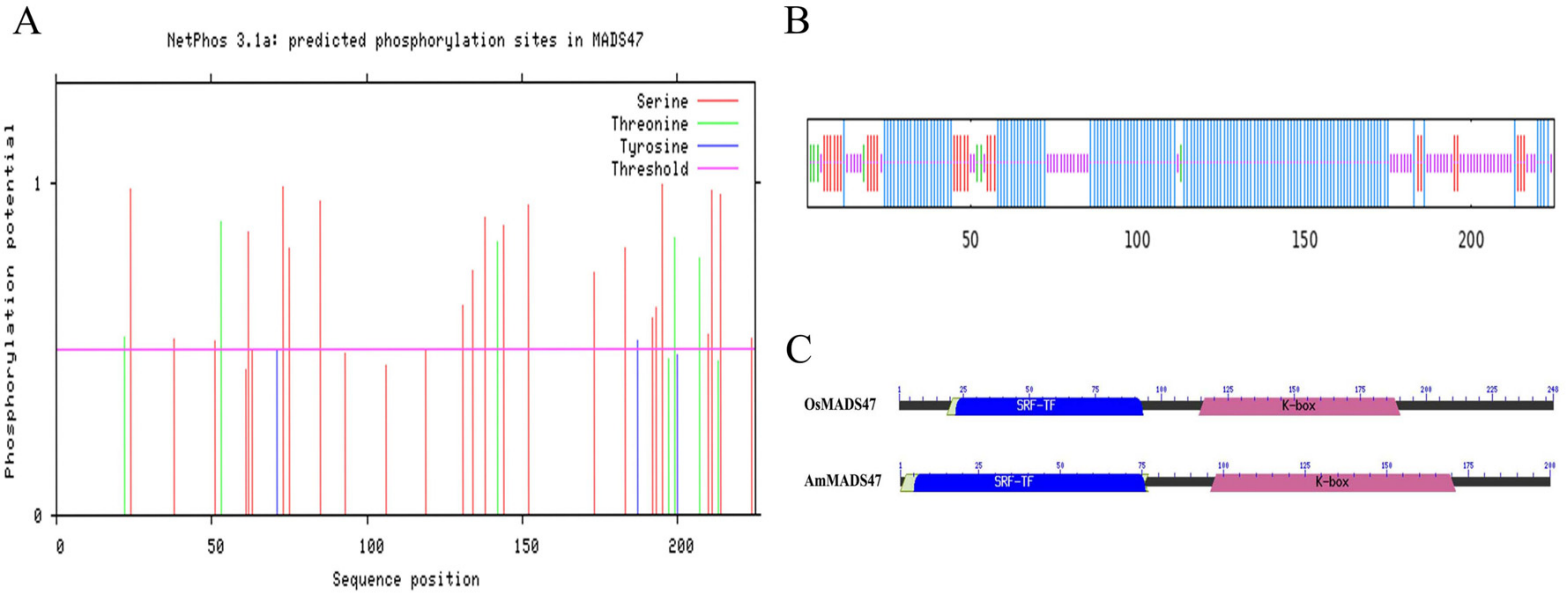
Basic bioinformatics analysis of *AmMADS47*. **(A)** Predictive analysis of phosphorylation sites in the *AmMADS47*. **(B)** Protein secondary structure of *AmMADS47*. **(C)** The protein structural domains of AmMADS47 and OsMADS47.

The coding sequence of the *AmMADS47* gene is 678 bp in length and encodes 225 amino acids (**Supplementary Figure S3**). Bioinformatics analysis was used to gain preliminary insight into the *AmMADS47* gene. The results showed significant homology between *AmMADS47* and rice’s *OsMADS47* gene (*LOC4331872*), with an 82% identity in Blast comparison (**Supplementary Figure S4A**). The phosphorylation sites in the AmMADS47 protein were primarily serine, threonine, and tyrosine (**Figure 1A**). Protein secondary and tertiary structures of AmMADS47 were characterized by alpha-helices and random coil (**Figures 1B**; **Supplementary Figure S4B**). Both AmMADS47 and OsMADS47 proteins contain SRF-TF and K-box structural domains, as indicated by structural domain analysis (**Figure 1C**).

**Figure 2:**
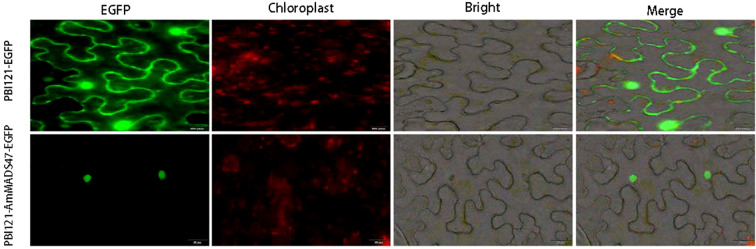
Subcellular localization of the *AmMADS47* protein. The scale of images PBI121-EGFP and PBI121-*AmMADS47*-EGFP is 50 μm.

Subcellular localization revealed that PBI121-EGFP localized to the cell membrane and nucleus, whereas PBI121-*AmMADS47*-EGFP located in the nucleus. This observation confirms nucleoprotein nature of the *AmMADS47* gene (**Figure 2**).

### 3.2 Impact of *AmMADS47* overexpression on drought tolerance in rice

**Figure 3:**
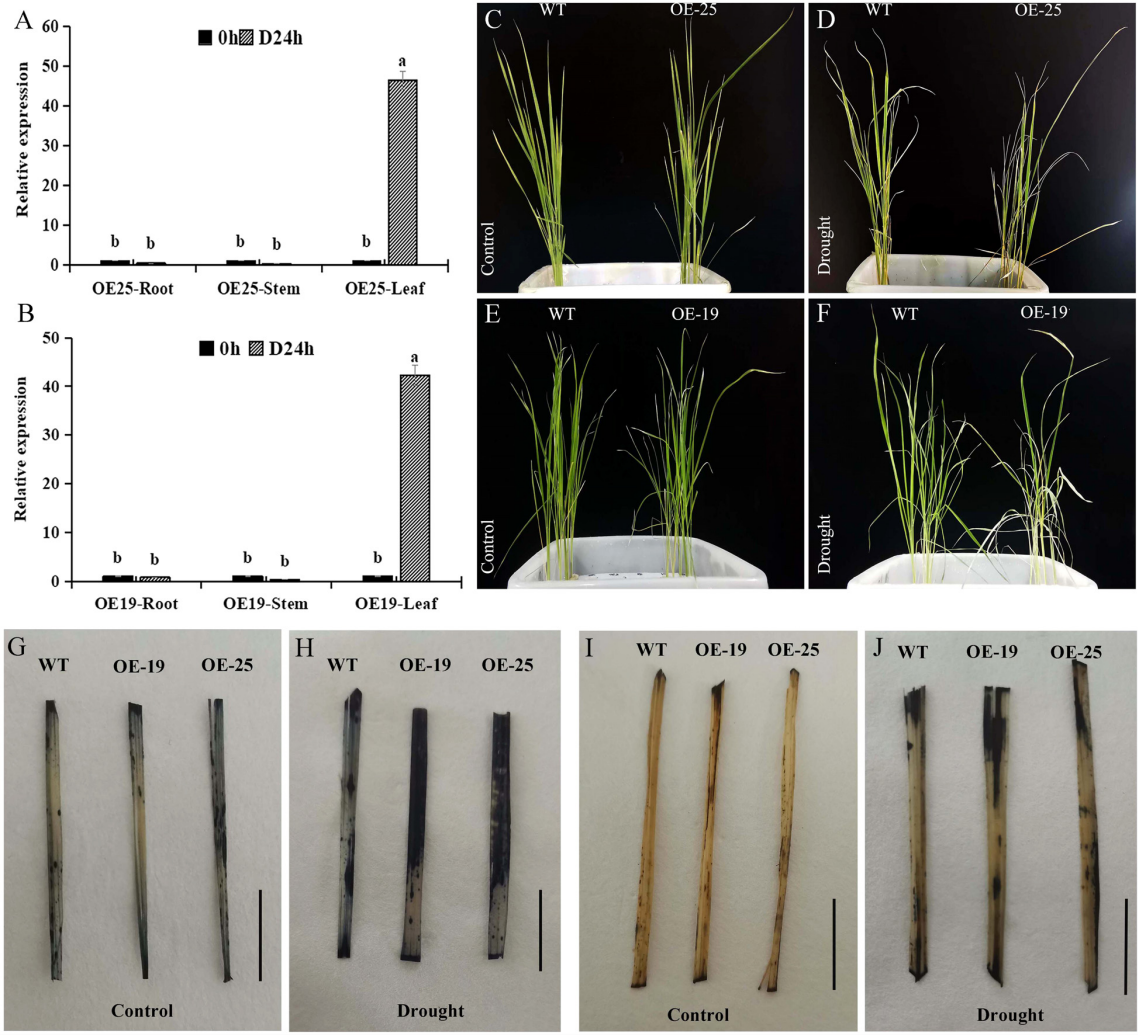
Analysis of *AmMADS47* expression and antioxidant capacity in rice. **(A, B)** Relative expression of *AmMADS47* in roots, stems and leaves of OE-25 and OE-19. Different letters in the column indicate a significant difference at the 0.05 level. **(C-F)** Phenotypes of OE-25 and OE-19 before and after osmotic stress. **(G, H)** NBT staining of leaves of WT, OE-19, and OE-25 before and after osmotic stress. **(I, J)** DAB staining of leaves of WT, OE-19, and OE-25 before and after osmotic stress. The scale in the image is 1 cm.

By means of the Agrobacterium-mediated transformation method for callus tissue, we obtained 41 transgenic rice plants (**Supplementary Figure S5**). The *AmMADS47* gene exhibited drought-induced up-regulation in rice, with higher relative expression observed in the OE-19 and OE-25 lines (**Supplementary Figure S6**). The drought-induced response of the *AmMADS47* gene was evident in leaves, as evidenced by qRT-PCR analysis of roots, stems, and leaves in the OE-19 and OE-25 lines (**Figures 3A, B**). These results showed that the *AmMADS47* gene was specifically expressed in rice leaves and responded to osmotic stress. Both OE-25 and OE-19 showed increased susceptibility to wilting and lodging compared to WT when observing phenotypes at the 4-leaf stage of rice before and after drought (**Figures 3C–F**).

Reactive oxygen species (ROS) accumulation is indicative of plant drought tolerance, with increased ROS levels corresponding to decreased tolerance. Superoxide anion (O^2-^) accumulation in WT, OE-19, and OE-25 was assessed through NBT staining. Before simulate drought stress, O^2-^ accumulation was lower in WT, OE-19, and OE-25 leaves (**Figure 3G**). However, after drought treatment, OE-19 and OE-25 showed significantly larger stained areas than WT, indicating increased O^2-^ accumulation in OE-19 and OE-25 leaves (**Figure 3H**). This suggested that overexpression of *AmMADS47* led to more severe oxidative damage under drought conditions. H_2_O_2_ accumulation in WT, OE-19, and OE-25 leaves was visualized through DAB staining. Before drought stress, H_2_O_2_ accumulation was lower in WT, OE-19, and OE-25 leaves (**Figure 3I**). Following osmotic stress, the staining area of OE-19 and OE-25 leaves was larger than that of WT (**Figure 3J**). These results showed that the overexpression of *AmMADS47* increased H_2_O_2_ accumulation in rice leaves, diminishing drought resistance.

**Figure 4:**
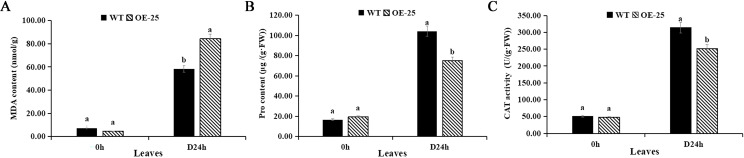
Analysis of drought related physiological indicators in OE-25. **(A)** MDA content of leaves of WT and OE-25 under osmotic stress. **(B)** Pro content of leaves of WT and OE-25 under osmotic stress. **(C)** CAT activity of leaves of WT and OE-25 under osmotic stress. Different letters on the column indicate significant difference at 0.05 level. Student T-test was used for the determination of significant differences.

Three physiological indexes related to drought resistance were measured in order to further understand the effects of overexpression of *AmMADS47* on the physiology of drought resistance in rice. After 24 hours of drought treatment, significant differences in the MDA content were found between WT and OE-25 leaves, measuring 84.21 and 58.18 nmol/g (**Figure 4A**). At the same time, the Pro content in OE-25 and WT leaves was 74.85 and 104.23 μg/(g·FW), respectively, suggesting a lower ability of OE-25 leaves to scavenge oxygen-free radicals compared to WT (**Figure 4B**). The CAT activity in WT and OE-25 leaves exhibited significant difference after 24 h of drought, with OE-25 and WT leaves recording 251.22 and 313.82 U/(g·FW), respectively. This indicates a weaker H_2_O_2_ decomposition ability in OE-25 compared to WT (**Figure 4C**).

### 3.3 Analysis of differentially expressed genes in trans-*AmMADS47* and wild-type rice

The raw data (NCBI ID: PRJNA954461) from transcriptome sequencing of 12 samples underwent filtering, resulting in clean bases ranging from 6.97 to 7.49 G, Q30 distribution from 95.04% to 96.24%, and GC content from 51.26% to 52.75% (**Supplementary Table S6**). The clean reads, when compared to the reference genome, fell within the range of 97.21% to 98.19%. The gene count for subsequent analysis, determined by htseq-count software, ranged from 26,905 to 27,755 (**Supplementary Table S7**). Principal component analysis (PCA) demonstrated good repeatability among biological samples (**Supplementary Figure S7A**). Hierarchical clustering divided samples into four branches (**Supplementary Figure S7B**), distinctly separating drought-treated samples (OED, WTD) from non-drought-treated samples (WT, OE).

**Figure 5:**
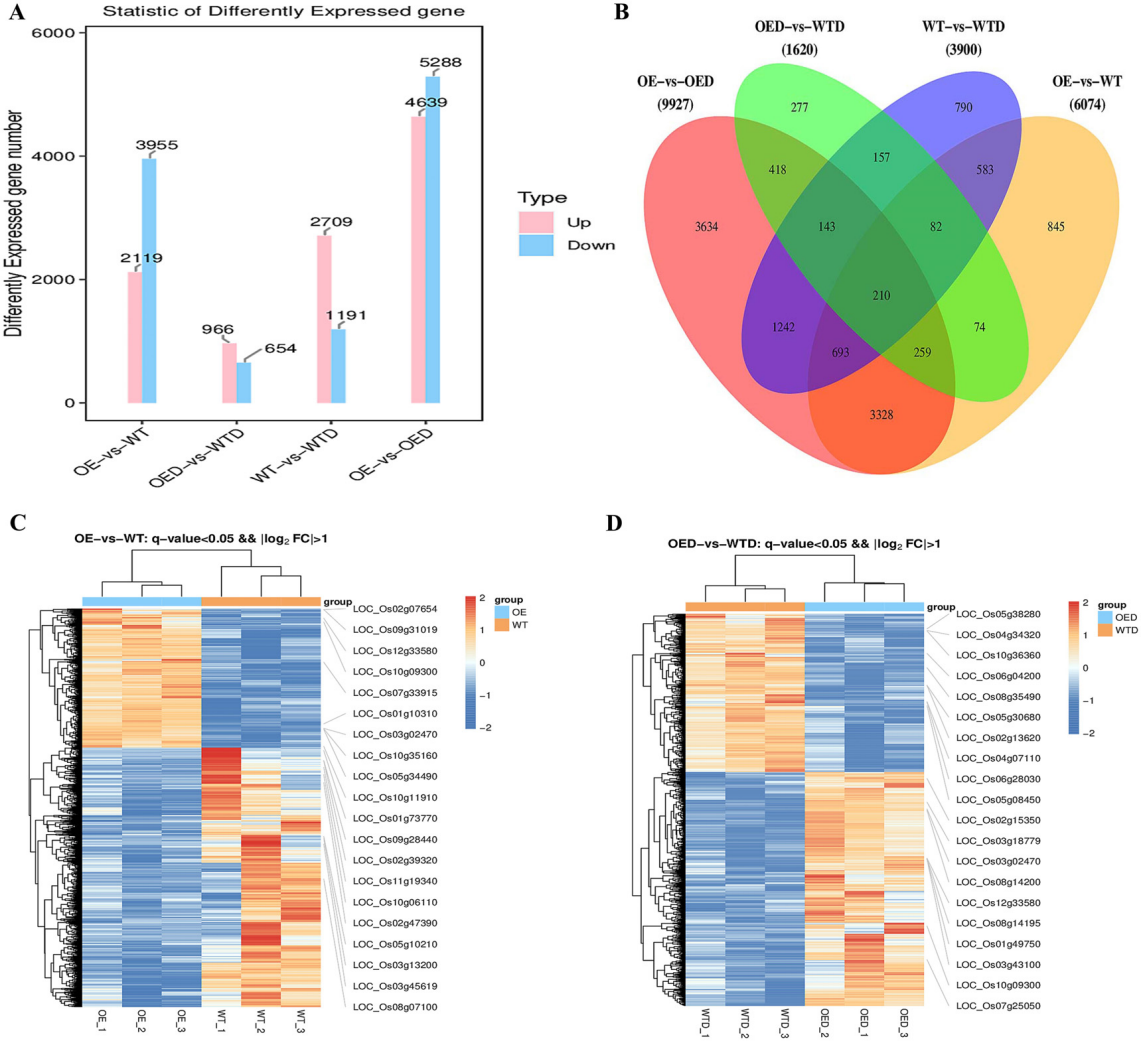
Statistical analysis and cluster heatmaps of DEGs. **(A)** Statistics on the number of up-regulated and down-regulated genes in DEGs, with pink indicating up-regulated genes and blue indicating down-regulated genes. **(B)** Statistical analysis of share and unique DEGs in different comparative groups. **(C, D)** Cluster heatmaps of DEGs from OE vs. WT and OED vs. WTD comparison groups; labeled on the right in the heatmap are the up-regulated and down-regulated top 10 DEGs. The expression level of genes in the heat map is the FPKM value.

DEGs in each comparison group were screened using *q* value and fold change (FC) threshold (**Supplementary Table S8**), resulting in 21,521 DEGs and 367 differentially expressed transcription factors. Analysis of up-regulated and down-regulated DEGs in the four comparison groups (OE vs. WT, OED vs. WTD, WT vs. WTD, and OE vs. OED) revealed varying counts. The number of up-regulated DEGs were 2,119, 966, 2,709, and 4,639, and the number of down-regulated DEGs were 3,955, 654, 1,191, and 5,288, respectively (**Figure 5A**). There were 210 genes shared among the four comparison groups. The specific 845 genes in OE vs. WT may be responsive to *AmMADS47* overexpression (**Figure 5B**), and 277 unique genes in OED vs. WTD may respond to *AmMADS47* overexpression and regulate rice osmotic stress. A cluster heat map showed distinct DEG expression patterns under overexpression and osmotic stress (**Figures 5C, D**). The hierarchical clustering heatmap, divided into two large branches, distinctly differentiated DEGs in the two comparison groups. OE exhibited significantly fewer up-regulated expressed genes than WT, suggesting that overexpression of *AmMADS47* resulted in a low-expression pattern in some DEGs.

**Figure 6:**
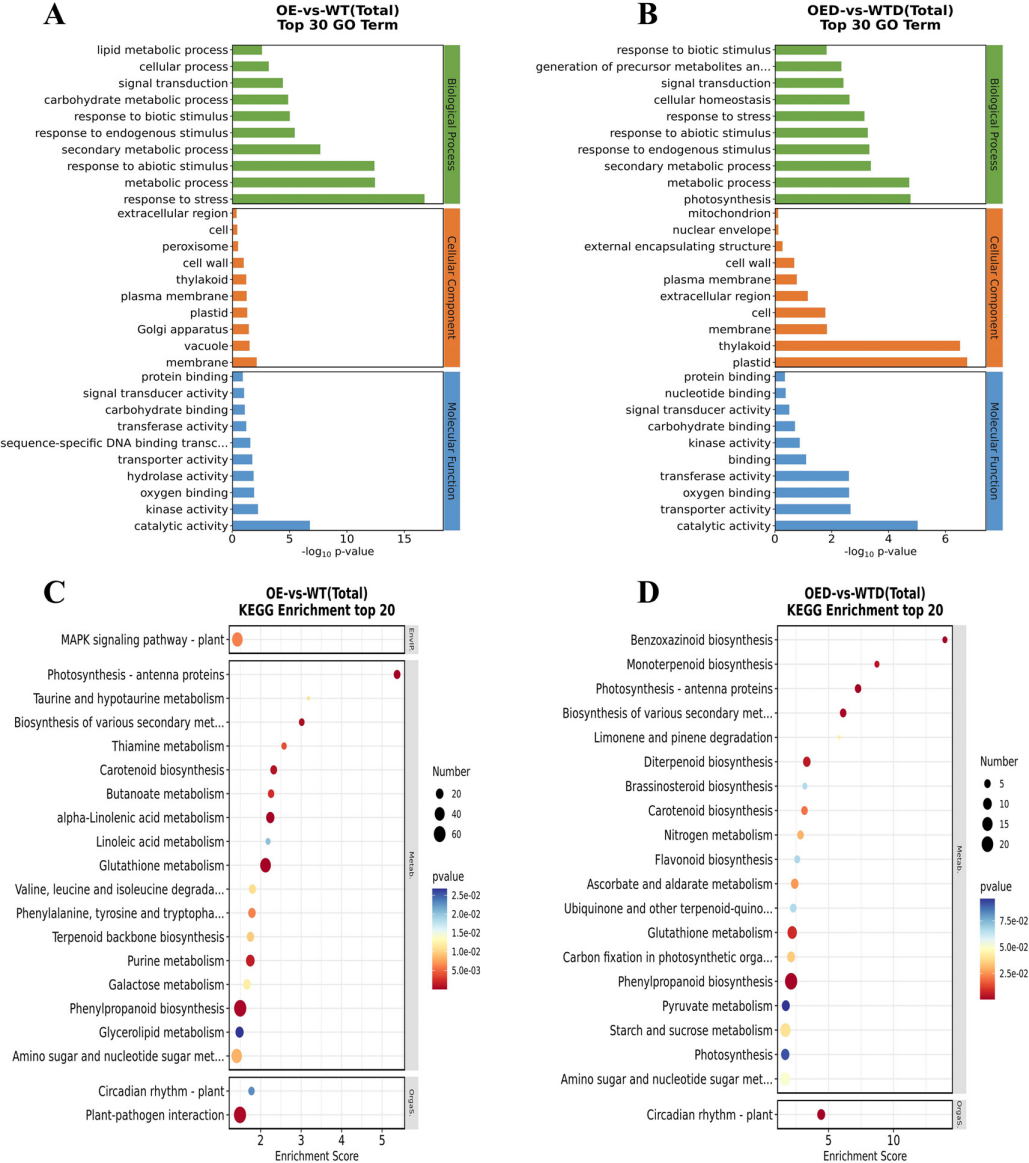
Top 30 GO and top 20 KEGG enrichment analysis of DEGs. **(A, B)** The top 30 GO terms in the comparison groups (OE vs. WT and OED vs. WTD). **(C, D)** The top 20 KEGG enrichment in the comparison groups (OE vs. WT and OED vs. WTD).

GO enrichment analysis of DEGs in the OE vs. WT and OED vs. WTD comparison groups revealed seven shared GO terms for biological processes, including signal transduction, response to biotic stimulus, response to endogenous stimulus, and others. For cellular components, seven shared GO terms, such as extracellular region, cell, cell wall, and others, were identified. In molecular function, eight shared GO terms, including protein binding, signal transducer activity, carbohydrate binding, and others, were found (**Figures 6A, B**). These shared GO terms may respond to *AmMADS47* overexpression and osmotic stress regulation. In the KEGG-enriched pathways analysis of the two comparison groups, phenylpropanoid biosynthesis and glutathione metabolism were identified as the shared pathways with the highest and the second-highest number of DEGs, respectively. The shared pathway with the highest enrichment scores and significance was photosynthesis-antenna proteins (**Figures 6C, D**). These three pathways were selected as candidate pathways for further analysis.

### 3.4 DEGs regulated to phenylpropanoid and lignin biosynthesis were important to drought stress

**Figure 7:**
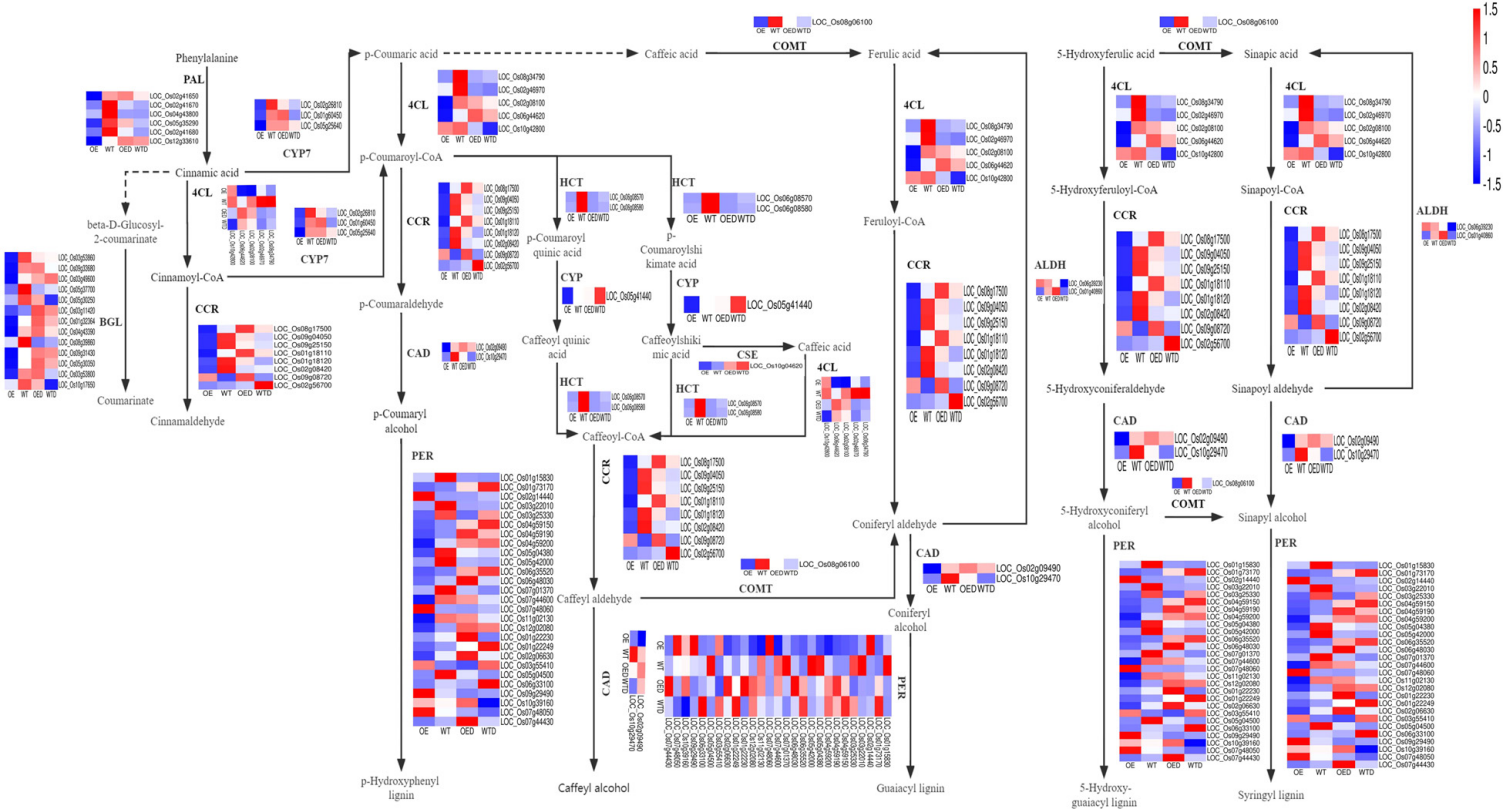
The phenylpropanoid biosynthesis pathway in two comparison groups (OE vs. WT, OED vs. WTD). This is a heatmap of the phenylpropanoid metabolism pathway and genes in two comparison groups. Blue indicates a down-regulation of gene expression levels, and red indicates an up-regulation.

In the phenylpropanoid biosynthesis pathway, there were 68 shared DEGs in both comparison groups (OE vs. WT and OED vs. WTD) (**Figure 7**). Key enzymes, such as phenylalanineammonia-lyase (PAL) and 4-coumarate coenzyme a ligase (4CL), determine lignin synthesis in phenylpropanoid biosynthesis pathway. Six genes regulating PAL showed down-regulation in OE compared to WT, suggesting a potential reduction in PAL synthesis. The drought regulation patterns of these six genes in wild-type rice were analyzed, revealing five genes (*LOC_Os02g41650, LOC_Os02g41670, LOC_Os04g43800, LOC_Os05g35290* and *LOC_Os02g41680*) were drought-sensitive, and one gene (*LOC_Os12g33610*) positively regulated drought. In OED, the expression of *LOC_Os12g33610* remained unchanged after osmotic stress, while four drought-sensitive genes (*LOC_Os02g41650*, *LOC_Os02g41670*, *LOC_Os05g35290*, and *LOC_Os02g41680*) were up-regulated compared to WTD, indicating that *AmMADS47* overexpression led to increased expression of most drought-sensitive genes regulating PAL, enhancing the drought sensitivity of overexpressed rice. For 4CL, five genes (*LOC_Os06g44620*, *LOC_Os08g34790*, *LOC_Os02g46790*, *LOC_Os02g08100*, and *LOC_Os10g42800)* were identified, all of which showed down-regulation in expression in OE compared to WT, potentially reducing the synthesis of 4CL. The analysis of their regulation model under osmotic stress in wild-type rice revealed four drought-sensitive genes (*LOC_Os08g34790*, *LOC_Os02g46790*, *LOC_Os02g08100*, and *LOC_Os10g42800*). After osmotic stress in OED, the expression of *LOC_Os02g08100* and *LOC_Os10g42800* genes was significantly up-regulated, while two genes showed no significant change compared to WTD. The overexpression of *AmMADS47* may contribute to a reduction in 4CL synthesis, and the up-regulated expression of drought-sensitive genes may reduce the drought resistance of transgenic rice.

In the lignin biosynthesis pathway, key downstream enzymes include cinnamoyl-coa reductase (CCR), cinnamyl alcohol dehydrogenase (CAD), hydroxycinnamoyl-CoA shikimate/quinate hydroxycinnamoyl transferase (HCT), and caffeate/5-hydroxyferulate 3-o-methyltransferase (COMT) (**Figure 7**). The expression of genes regulating these enzymes is mostly down-regulated in OE compared to in WT, potentially resulting in reduced synthesis of these important enzymes. For example, 8 genes regulating CCR, seven of which are down-regulated in OE compared to WT, and 2 genes (*LOC_Os02g09490* and *LOC_Os10g29470*) regulating CAD, both showing down-regulation for gene expression in OE compared to WT (**Figure 7**). The regulation mode of these pathway genes under osmotic stress was analyzed. Drought-sensitive genes in the wild type were up-regulated in OED compared to WTD, indicating an increase in drought sensitivity in overexpressed rice. For example, among the genes regulating CCR, three drought-sensitive genes (*LOC_Os09g04050*, *LOC_Os09g25150*, and *LOC_Os01g18120*) in the wild-type rice were up-regulated in OED compared with WTD, while the expression of *LOC_Os02g08420* showed no significant change. The two genes (*LOC_Os02g09490* and *LOC_Os10g29470)* regulating CAD, which were drought-sensitive in the wild-type rice, were up-regulated in OED after osmotic stress compared with WTD. These results showed that overexpression of *AmMADS47* reduced the expression of genes regulating important enzymes in the phenylpropanoid biosynthesis pathway, leading to impaired lignin synthesis. Additionally, of the up-regulation of drought-sensitive genes in overexpressed rice contributes to a reduction in drought resistance.

### 3.5 DEGs involved in glutathione metabolism were responsive to drought stress

**Figure 8:**
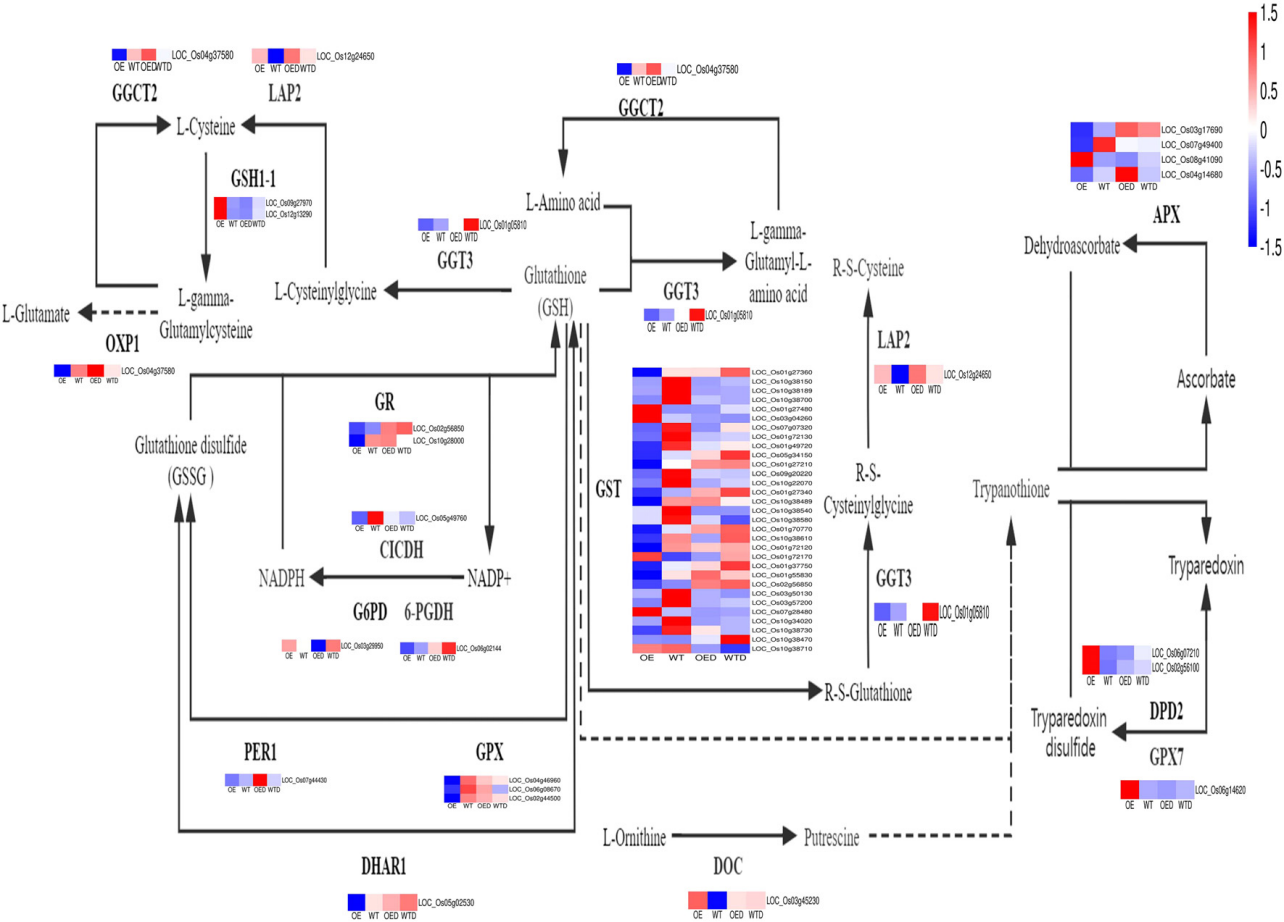
The glutathione metabolism pathway in two comparison groups (OE vs. WT, OED vs. WTD). This is a heatmap of the glutathione metabolism pathway and genes in two comparison groups. Blue indicates a down-regulation of gene expression levels, and red indicates an up-regulation.

The share of DEGs in the glutathione metabolism pathway in the two comparison groups (OE vs. WT and OED vs. WTD) were 58 (**Figure 8**). Key enzymes in the glutathione metabolism pathway include glutathione transferases (GSTs), ascorbate peroxidase (APX), and glutathione peroxidase (GPX). The decrease in the expression of three enzymes can reduce the resistance of plants to ROS damage. There are 31 genes regulating GST, such as *LOC_01g27360*, *LOC_10g38150*, and *LOC_10g38189* and others. Among these, 26 genes were down-regulated in OE compared to gene expression in WT, potentially reducing the synthesis of GST. Among the 26 genes, 17 were drought-sensitive, and 9 were positively regulated by drought stress in wild-type rice. After simulate drought stress, two drought-sensitive genes (*LOC_07g07320*, *LOC_01g49720*) were down-regulated in OED compared with WTD, indicating that overexpressed rice is more sensitive to drought. Three drought-sensitive genes (*LOC_10g38489*, *LOC_10g38580*, and *LOC_10g38730*) were up-regulated, reducing the drought resistance of transgenic rice. The other 11 genes showed insignificant changes in expression in WTD and OED. After simulate drought stress, 8 genes positively regulated by drought were down-regulated in OED compared with the situation in WTD. It indicated that overexpression of *AmMADS47* decreased the expression of most of the positively regulated drought genes, thus reducing the drought resistance of overexpressed rice. There were four genes that regulate APX (*LOC_03g17690*, *LOC_07g49400*, *LOC_04g14680*, and *LOC_08g41090*), and three of them (*LOC_03g17690*, *LOC_07g49400*, and *LOC_04g1468*) were down-regulated in OE compared to gene expression in WT, potentially decreasing APX synthesis. *LOC_08g41090* gene was positively regulated by drought stress in wild-type rice. After osmotic stress, its expression was down-regulated in OED compared with WTD, reducing drought tolerance in transgenic rice. For GPX, three genes (*LOC_04g46960*, *LOC_06g08670*, and *LOC_02g44500*) were down-regulated in OE compared with WT, potentially reducing GPX synthesis. These three genes were drought-sensitive in wild-type rice. After osmotic stress, their expression was up-regulated in OED compared to WTD, decreasing the drought resistance of overexpressed rice. The overall analysis of the glutathione metabolism pathway suggested that overexpression of *AmMADS47* could reduce the expression of genes regulating key enzymes, leading to a decrease in the synthesis of these enzymes. Most genes positively regulating drought were down-regulated, while most drought-sensitive genes were up-regulated for expression, collectively reducing the drought resistance of rice overexpressing *AmMADS47*.

### 3.6 ATP anabolism related DEGs were participated in the response to drought stress

**Figure 9:**
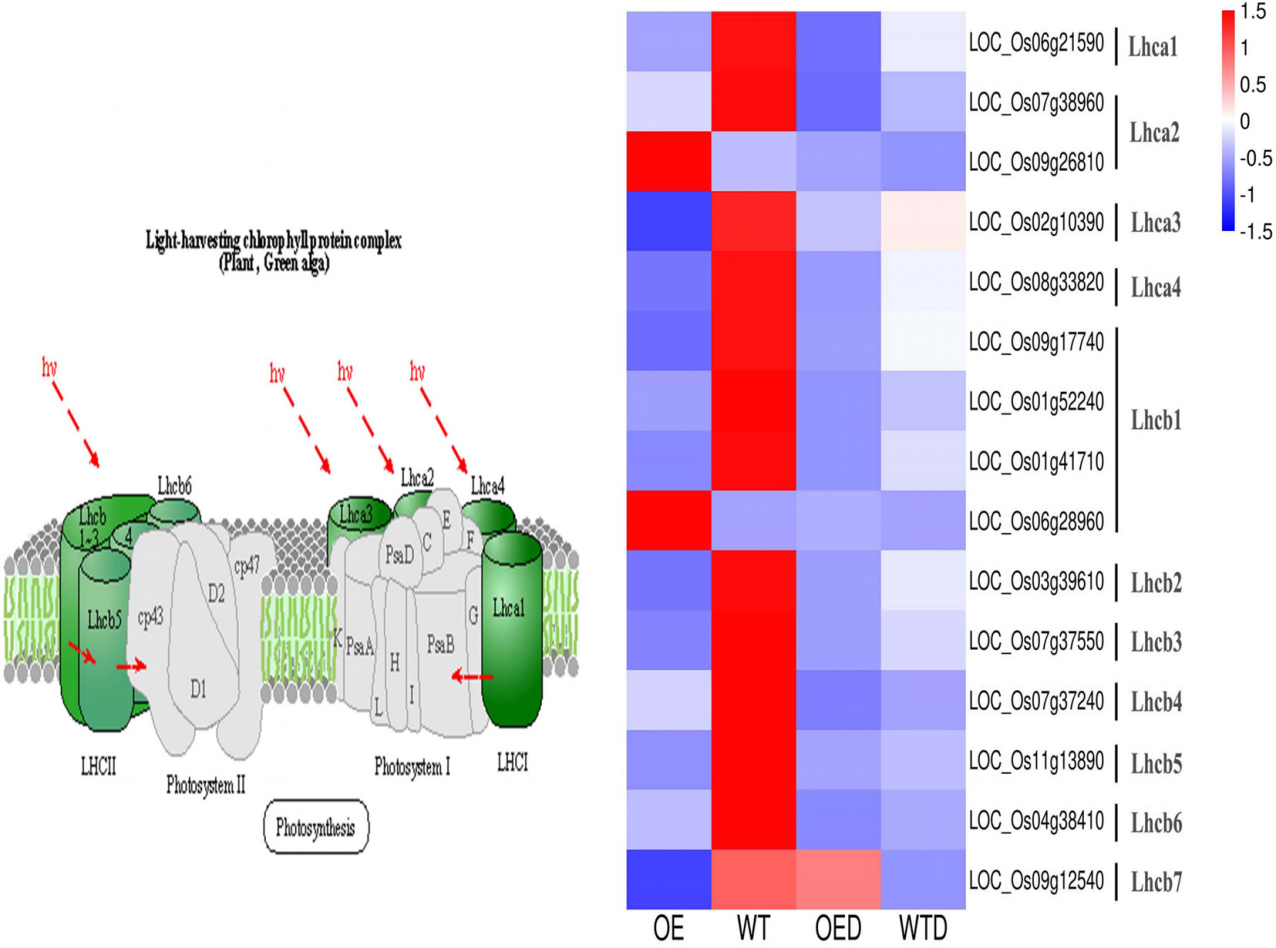
The photosynthesis-antenna proteins pathway in two comparison groups (OE vs. WT, OED vs. WTD). This is a heatmap of the photosynthesis-antenna proteins pathway and genes in two comparison groups. Blue indicates a down-regulation of gene expression levels, and red indicates an up-regulation.

The energy used for the growth and development of higher plants comes mainly from adenosine triphosphate (ATP) produced by photosynthesis, and chloroplasts is the main source of plant photosynthesis. Drought stress affects the photosynthesis-antenna proteins pathway and regulates the expression of light-harvesting complexes proteins (LHCs). In the photosynthesis-antenna proteins pathway, 15 shared genes were annotated in the two comparison groups (OE vs. WT, OED vs. WTD), and these 15 genes were further annotated into 11 LHCs. The expression of 13 genes in OE was decreased compared to WT, indicating a potential reduction in the synthesis of LHCs (**Figure 9**). These 13 genes were identified as drought-sensitive genes in wild-type rice. After osmotic stress, the expression of 12 genes in OED was lower than that in WTD. However, the expression of *LOC_Os09g12540* was higher in OED than in WTD. These changes may collectively contribute to a decrease in drought tolerance in overexpressed rice. Additionally, the expression of two genes (*LOC_Os09g26810* and *LOC_Os06g28960*) was up-regulated in OE compared to WT. However, the expression of these two genes did not change significantly in OED and WTD after osmotic stress. It can be speculated that these two genes may not actively participate in the regulation of osmotic stress under the condition tested.

**Figure 10:**
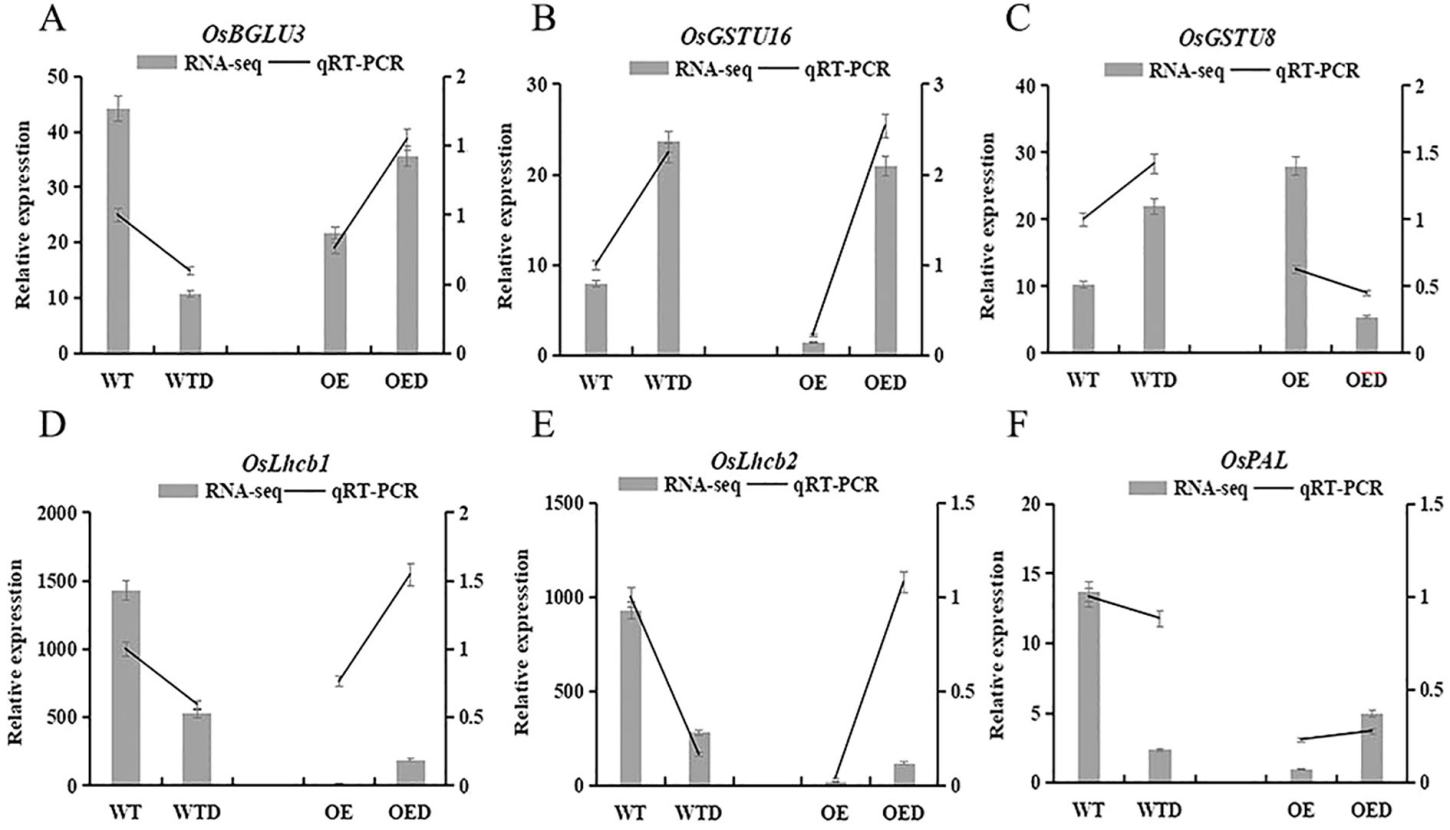
Expression analysis of annotation genes in three candidate pathways of rice. OsBGLU3, OsGSTU16, OsGSTU8, OsLhcb1, OsLhcb2, and OsPAL.

Six genes involved in the phenylpropanoid biosynthesis, glutathione metabolism, and the photosynthesis-antenna proteins pathway were selected for qRT-PCR analysis, and the relative gene expression trends observed were consistent with the transcriptome sequencing (**Figure 10**). This alignment indicated that the rice transcriptome sequencing data were accurate and reliable. Furthermore, the expression trends of five genes in wild-type rice were opposite to those in overexpressed rice. *OsGSTU16* exhibited a similar expression trend in both wild-type rice and overexpressed rice, but its expression was downregulated in the overexpressed rice compared to wild-type rice. This suggested that overexpression of the *AmMADS47* gene may change the expression pattern of certain genes in these three pathways, consequently influencing the drought resistance of rice.

## 4 Discussion

### 4.1 Functional analysis of the *AmMADS47*

MADS-box transcription factors exert crucial regulatory functions in plants’ response to abiotic adversity stress. Currently, there is limited research on the *MADS47* gene, particularly regarding its regulatory functions. Existing studies in rice have enhanced the negative regulation of the brassinolide signal transduction pathway by the *OsMADS47* gene (Lee et al., 2008b; Duan et al., 2010). However, there is a notable gap in understanding the *AmMADS47* gene’s involvement in drought regulation in *A. mongolicum*. In response to drought conditions, *AmMADS47* is induced and actively participates in the intricate network governing drought stress. Unraveling the functional role of this gene holds significant importance. In this study, AmMADS47 protein was localized in the nucleus. The investigation extended to assess the tissue-specific expression of *AmMADS47* in overexpressed rice roots, stems, and leaves. Notably, *AmMADS47* exhibited significant expression specificity in leaves following simulate drought stress. Observing the phenotype of rice revealed that, under osmotic stress, OE-19 and OE-25 displayed enhanced wilting compared to WT, accompanied by curled leaves and lodging. This result is consistent with the study of Khong et al, who proved that *OsMADS26* negatively influences drought resistance in rice (Khong et al., 2015). Similar to our results, over-expressed *OSMADS26* in rice resulted in a decrease in drought tolerance and the manifestation of several adversity phenotypes, such as slow root and branch growth and the development of light green plants (Khong et al., 2015). Further assessments using NBT and DAB staining to visualize ROS damage demonstrated that OE-25 and OE-19 suffered more significant ROS damage than WT. This observation was consistent with the results of MDA, Pro, and CAT determinations, emphasizing the negative regulatory impact of *AmMADS47* overexpression on rice drought resistance. AmMADS47 interact with proteins of MAPK family (**Supplementary Figure S8**), which have been proved to participate in drought response through regulating ROS signaling. MAPK cascades are involved in ROS signal transduction, which is induced by ROS signaling and can regulate the production of ROS (Zhang et al., 2014). Published studies show that MPK2 is involved in regulating stomatal opening (Li et al., 2023), MPK5 is regulated by oxidative stress, MPK6 in *Arabidopsis* is activated under abiotic stress, and participates in stomatal morphology, redox regulation and hormone regulation (Colcombet and Hirt, 2008; Zhang et al., 2014); MPK8 negatively regulates ROS accumulation by controlling *Rboh D* gene (Takahashi et al., 2011). So, we think *AmMADS47* regulates the drought resistance of rice by influencing the ROS pathway, too.

### 4.2 Effects of overexpression of *AmMADS47* in rice on downstream genes

Utilizing RNA-seq analysis, downstream response genes and pathways specific to genetic transformation events can be elucidated. For instance, the introduction of the *GhSAMDC1* gene into tobacco resulted in the identification of 938 differentially responsive genes through RNA-seq analysis. Comprehensive regulatory networks governing nutritional growth and early flowering in both wild-type and transgenic tobacco were unveiled by employing GO and KEGG analysis (Cheng et al., 2022). Similarly, an exploration of the molecular mechanisms underlying leaf roll phenotypes in overexpressed rice lines was conducted via RNA-seq. In this context, 2,920 differentially responsive genes, 42 belonged to the KEGG pathway (dosa04075), were identified (Yu et al., 2022). Applying a similar approach, the downstream genes and pathways responsive to *AmMADS47* in rice were investigated using RNA-seq. Sequencing the transcriptomes of *AmMADS47-*overexpressing rice and wild-type rice leaves allowed for the analysis of *AmMADS47*’s impact on rice drought tolerance. Large numbers of genes were differentially expressed between *AmMADS47*-overexpressing rice and wild-type rice leaves. These DEGs were enriched in the biological processes of signal transduction, response to biotic stimulus, response to endogenous stimulus, and others. Moreover, the analysis of KEGG pathways highlighted that phenylpropanoid biosynthesis and glutathione metabolism as shared pathways, with photosynthesis-antenna proteins ranking highest in enrichment score and significance. Our results also proved that DEGs regulated to ATP anabolism were important to drought stress, these results imply the rapid response of secondary metabolites under abiotic stress, and a similar response mechanism also existed in cotton roots and leaves (Su et al., 2020; Nie et al., 2021).

### 4.3 Some metabolic pathways are important to drought tolerance

In this study, 68 shared annotated genes were identified in the phenylpropanoid biosynthesis pathway. The expression of genes regulating PAL was down-regulated in OE compared to WT. Most genes involved in the regulation of 4CL, CCR, CAD, HST, and COMT exhibited a similar down-regulated as PAL in OE, potentially diminishing the synthesis of key enzymes and, consequently, affecting the synthesis of lignin and reducing the drought resistance of rice. Phenylpropanoid metabolism is crucial in regulating abiotic stress responses in crops, including drought, low temperature, and high temperature, contributing to enhanced crop resistance (Cisneros-Zevallos, 2003; Marie et al., 2003; Hano et al., 2006). Comprising lignin and flavonoid biosynthetic pathways, the phenylpropanoid biosynthetic pathway is pivotal for plant stress adaptation. Lignin is a vital component of the cell wall, contributes to maintaining cellular osmotic balance, influencing plant resistance to lodging, drought, diseases, and pests (Moura et al., 2010), allogenic expression *OsGRP3* in rice modulates lignin accumulation and improves drought resistance (Xu et al., 2022). They has demonstrated that some crucial enzymes, such as 4CL, CCR, CAD, and COMT, participated in the lignin synthesis pathway and responded to drought stress (Hu et al., 2009; Leyva et al., 1995), and similar results were demonstrated in our study in leaves of rice.

Adverse stress conditions trigger the production of ROS in plants, necessitating the activation of plant antioxidant systems to counteract ROS accumulation. The glutathione metabolic pathway, an important player in the scavenging of ROS in plants, prominently features antioxidant enzymes such as APX, GPX, and GST. In this study, 58 genes were annotated to the glutathione metabolic pathway, with APX, GPX, and GST identified as key enzymes. Among these, 4, 3, and 31 genes were involved in regulating APX, GPX, and GST, respectively. The majority of these genes exhibited lower expression levels in OE compared to WT. Previous studies have proved that GPX catalyzes the reduction of H_2_O_2_ and protects plants from oxidative damage (Arthur, 2001). The knockout of the *AtGPX3* gene in *Arabidopsis thaliana* resulted in a significant decrease in drought resistance and antioxidant capacity (Kang et al., 2004). Enhancing the expression of GST gene has been shown to improve the ability of plants to scavenge ROS, thereby improving drought tolerance. Overexpression of *ThGSTZ1* from *Tamarix hispida* in *Arabidopsis* led to increased activities of GST and glutathione peroxidase, a decrease in ROS levels, and increased drought resistance (Yang et al., 2014). All of the above results show that a variation in the synthesis of APX, GPX, and GST ultimately changes the drought resistance of different plants.

Photosynthesis serves as the primary source of energy in plants, with light capture being a fundamental prerequisite for this process. The regulation of LHCs gene expression directly affected protein synthesis, thus impacts photosynthesis. Drought stress exerted a significant effect on photosynthesis, as evidenced by the advanced peak value of daily changes in the photosynthetic rate observed in maize under drought stress, facilitating normal photosynthesis despite the adverse conditions (Yu et al., 2015). The modulation of LHCs gene expression played a crucial role in chlorophyll synthesis, a key component of photosynthesis. Knockout of the *OsLHCB3* gene in rice resulted in light green leaves during tillering and heading stages, accompanied by a reduction in chlorophyll content (Wang et al., 2023). Similarly, *Arabidopsis* leaves lacking the *Lhcb1* and *Lhcb2* genes exhibited a light green phenotype and decreased chlorophyll content (Jenny et al., 2003). In the present study, 15 genes were annotated in the photosynthesis-antenna proteins pathway, regulating 11 LHCs. Strikingly, the expression of 13 genes in OE was lower than that in WT, suggesting a potential reduction in the synthesis of LHCs and chlorophyll. This decrease in energy supply may compromise plant photosynthesis, leading to reduced drought resistance in *AmMADS47*-overexpressing rice.

## Conclusion

**Figure 11:**
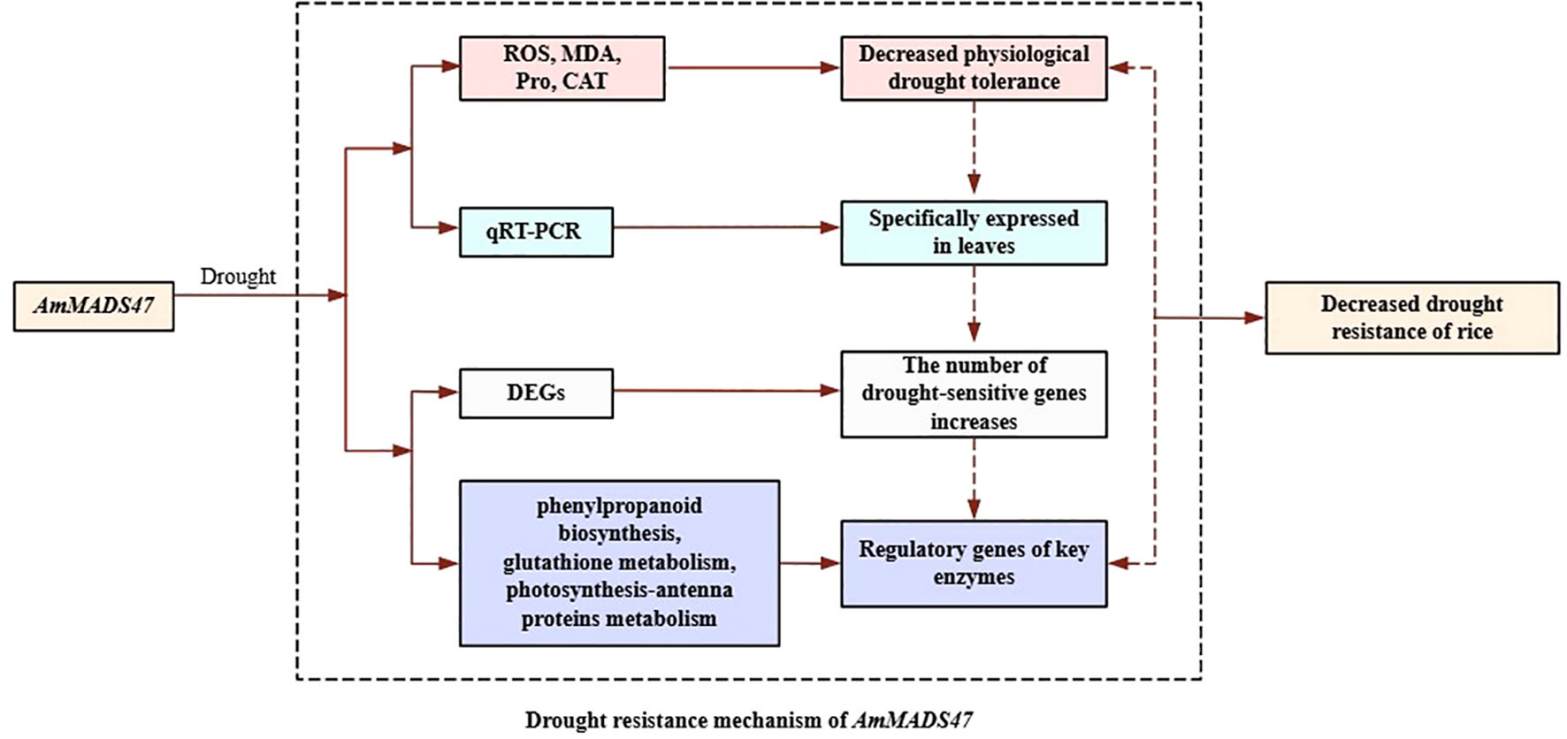
Drought resistance mechanism of *AmMADS47*.

This study elucidates the molecular mechanism whereby *AmMADS47* overexpression compromises drought tolerance in rice (**Figure 11**). Transgenic plants exhibited pronounced growth impairment under simulate drought stress, characterized by excessive ROS accumulation, reduced CAT activity, elevated MDA content, and reduced proline levels, proved that *AmMADS47* may be involved in regulation of drought tolerance in transgenic rice by influencing the ROS pathway. In addition, transcriptome sequencing analysis found that *AmMADS47* overexpression affected the gene expression of key enzymes in three candidate metabolic pathways (phenylpropanoid biosynthesis, glutathione metabolism and photosynthesis-antenna proteins), and it was speculated that it affected the synthesis of key enzymes and reduced the drought resistance of rice. These findings establish a foundation for the drought-responsive functions of *AmMADS47*. Future investigations will systematically examine *AmMADS47*-mediated transcriptional regulation of downstream targets and associated metabolic reprogramming mechanisms, facilitating the exploitation of wild genetic resources for crop drought resistance improvement.

## Data Availability

The datasets presented in this study can be found in online repositories. The names of the repository/repositories and accession number(s) can be found below: https://www.ncbi.nlm.nih.gov/genbank/, PRJNA954461.

## Supplementary material

Supplementary Figure 1 Positive identification of *AmMADS47* overexpression vector.

Supplementary Figure 2 Genetic transformation of rice.

Supplementary Figure 3 Comparison analysis of AmMADS47 and protein secondary structure.

Supplementary Figure 4
*AmMADS47* CDS translation.

Supplementary Figure 5 Positive identification of rice.

Supplementary Figure 6 Relative expression of overexpression rice leaves.

Supplementary Figure 7 Repeatability and hierarchical clustering analysis of samples.

Supplementary Figure 8 AmMADS47 protein interaction prediction.

## References

[B1] Abdullah-Zawawi M. Ahmad-Nizammuddin N. Sarahani Harun N. Mohd-Assaad N. Mohamed-Hussein Z. (2021). Comparative genomewide analysis of WRKY, MADS-box and MYB transcription factor families in *Arabidopsis* and rice. Sci. Rep-UK. 11, 19678–19695. doi: 10.1038/s41598-021-99206-y PMC8490385 34608238

[B2] Aglawe S. B. Fakrudin B. Patole C. B. Bhairappanavar S. B. Koti R. V. Krishnaraj P. U. (2012). Quantitative RT-PCR analysis of 20 transcription factor genes of MADS, ARF, HAP2, MBF and HB families in moisture stressed shoot and root tissues of sorghum. Physiol. Mol. Biol. Pla. 18, 287–300. doi: 10.1007/s12298-012-0135-5 PMC3550552 24082491

[B3] Arthur J. R. (2001). The glutathione peroxidase. Cell. Mol. Life Sci. 57, 1825–1835. doi: 10.1007/PL00000664 PMC11147127 11215509

[B4] Becker A. Theißen G. (2003). The major clades of MADS-box genes and their role in the development and evolution of flowering plants. Mol. Phylogenet. Evol. 29, 464–489. 14615187 10.1016/s1055-7903(03)00207-0

[B5] Boyer J. S. (1982). Plant productivity and environment (crop genetic improvement). Science 218, 443–448. doi: 10.1126/science.218.4571.443 17808529

[B6] Chandra P. Wunnava A. Verma P. Chandra A. Sharma R. K. (2021). Strategies to mitigate the adverse effect of drought stress on crop plants-influences of soil bacteria: A review. Pedosphere 31, 496–509. doi: 10.1016/S1002-0160(20)60092-3

[B7] Chen S. Zhou Y. Chen Y. Jia G. (2018). fastp: an ultra-fast all-in-one FASTQ preprocessor. Bioinformatics 34, 884–890. doi: 10.1093/bioinformatics/bty560 30423086 PMC6129281

[B8] Cheng X. Pang F. Tian W. Tang X. Wu L. Hu X.. (2022). Transcriptome analysis provides insights into the molecular mechanism of *GhSAMDC1* involving in rapid vegetative growth and early flowering in tobacco. Sci. Rep-UK. 12, 13612–13623. doi: 10.1038/s41598-022-18064-4 PMC9365820 35948667

[B9] Cisneros-Zevallos L. (2003). The use of controlled postharvest abiotic stresses as a tool for enhancing the nutraceutical content and adding-value of fresh fruits and vegetables. J. Food Sci. 68, 1560–1565. doi: 10.1111/j.1365-2621.2003.tb12291.x

[B10] Colcombet J. Hirt H. (2008). Arabidopsis MAPKs: a complex signaling network involved in multiple biological processes. Biochem. J. 413, 217–226. doi: 10.1042/BJ20080625 18570633

[B11] Du J. Li X. Li T. Yu D. Han B. (2017). Genome-wide transcriptome profiling provides overwintering mechanism of *Agropyron mongolicum* . BMC Plant Biol. 17, 1–13. doi: 10.1186/s12870-017-1086-3 28797236 PMC5553669

[B12] Duan K. Li L. Hu P. Xu S. P. Xu Z. H. Xue H. W. (2010). A brassinolide-suppressed rice MADS-box transcription factor, *OsMDP1*, has a negative regulatory role in BR signaling. Plant J. 47, 519–531. doi: 10.1111/j.1365-313X.2006.02804.x 16827922

[B13] Esfahanian E. Nejadhashemi A. P. Abouali M. Adhikari U. Zhang Z. Daneshvar F.. (2017). Development and evaluation of a comprehensive drought index. J. Environ. Manage. 185, 31–43. doi: 10.1016/j.jenvman.2016.10.050 28029478

[B14] Fan B. Sun F. Yu Z. Zhang X. Yu X. Wu J. (2022). Integrated analysis of small RNAs, transcriptome and degradome sequencing reveal the drought stress network in *Agropyron mongolicum* Keng. Fronts. Plant Sci. 13. doi: 10.3389/fpls.2022.976684 PMC9433978 36061788

[B15] Fornara F. Gregis V. Pelucchi N. Colombo L. Kater M. (2008). The rice *StMADS11*-*like* genes *OsMADS22* and *OsMADS47* cause floral reversions in *Arabidopsis* without complementing the svp and agl24 mutants. J. Exp. Bot. 59, 2181–2190. doi: 10.1093/jxb/ern083 18453531 PMC2413287

[B16] Han Y. Yang R. Zhang X. Wang Q. Wang B. Zheng X.. (2022). Brassinosteroid accelerates wound healing of potato tubers by activation of reactive oxygen, metabolism and phenylpropanoid metabolism. Foods 11, 906–921. doi: 10.3390/FOODS11070906 35406993 PMC8997868

[B17] Hano C. Addi M. Bensaddek L. Cronier D. Baltora-Rosset S. Doussat J.. (2006). Differential accumulation of monolignol-derived compounds in elicited flax (*Linum usitatissimum*) cell suspension cultures. Planta 223, 975–989. doi: 10.1007/s00425-005-0156-1 16292660

[B18] Hu Y. Li W. Xu Y. Li G. Liao Y. Fu F. (2009). Differential expression of candidate genes for lignin biosynthesis under drought stress in maize leaves. J. Appl. Genet. 50, 213–223. doi: 10.1007/BF03195675 19638676

[B19] Huang J. Yu H. Guan X. Wang G. Guo R. (2015). Accelerated dryland expansion under climate change. Nat. Climate Change 6, 166–171. doi: 10.1038/nclimate2837

[B20] Jenny A. Mark W. Robin G. W. Caroline A. H. Alexander V. R. Peter H.. (2003). Absence of the Lhcb1 and Lhcb2 proteins of the light-harvesting complex of photosystem II effects on photosynthesis, grana stacking and fitness. Plant J. 35, 350–361. doi: 10.1046/j.1365-313x.2003.01811.x 12887586

[B21] Jiao H. Hua Z. Zhou J. Hu J. Zhao Y. Wang Y.. (2023). Genome-wide analysis of Panax MADS-box genes reveals role of *PgMADS41* and *PgMADS44* in modulation of root development and ginsenoside synthesis. Int. J. Biol. Macromol. 233, 1–48. doi: 10.1016/j.ijbiomac.2023.123648 36780966

[B22] Kang S. Jeong K. Suh S. (2004). Characterization of a new member of the glutathione peroxidase gene family in *Oryza sativa* . Mol. Cells 17, 23–28. doi: 10.1242/jcs.00946 15055522

[B23] Khong G. N. Pati P. K. Richaud F. Parizot B. Bidzinski P. Mai C. D.. (2015). *OsMADS26* negatively regulates resistance to pathogens and drought tolerance in rice. Plant Physiol. 169, 2935–2949. doi: 10.1104/pp.15.01192 26424158 PMC4677910

[B24] Kim D. Langmead B. Salzberg S. (2015). HISAT: a fast spliced aligner with low memory requirements. Nat. Methods 12, 357–360. doi: 10.1038/nmeth.3317 25751142 PMC4655817

[B25] Lee S. Choi S. C. An G. (2008b). Rice SVP-group MADS-box proteins, *OsMADS22* and OsMADS55, are negative regulators of brassinosteroid responses. Plant J. 54, 93–105. doi: 10.1111/j.1365-313X.2008.03406.x 18182025

[B26] Lee S. Woo Y. Ryu S. Shin Y. Kim W. Park Y.. (2008a). Further characterization of a rice *AGL12* group MADS-box gene, *OsMADS26* . Plant Physiol. 147, 156–168. doi: 10.1007/s00438-014-0912-7 18354041 PMC2330315

[B27] Lesk C. Rowhani P. Ramankutty N. (2016). Influence of extreme weather disasters on global crop production. Nature 529, 84. doi: 10.1038/nature16467 26738594

[B28] Leyva A. Jarillo J. Julio S. Martínez-Zapater J. (1995). Low temperature induces the accumulation of phenylalanine ammonia-Lyase and chalcone synthase mRNAs of *Arabidopsis thaliana* in a light-dependent manner. Plant Physiol. 108, 39–46. doi: 10.1093/jxb/ern152 12228452 PMC157303

[B29] Li F. Chen X. Zhou S. Xie Q. Wang Y. Xiang X.. (2020). Overexpression of *SlMBP22* in tomato affects plant growth and enhances tolerance to drought stress. Plant Sci. 301, 110672. doi: 10.1016/j.plantsci.2020.110672 33218637

[B30] Li Y. Hui S. Yuan Y. Ye Y. Ma X. Zhang X.. (2023). PhyB-dependent phosphorylation of mitogen-activated protein kinase cascade MKK2-MPK2 positively regulates red light-induced stomatal opening. Plant Cell Environ. 46, 3323–3336. doi: 10.1111/pce.14675 37493364

[B31] Li X. Yu B. Wu Q. Min Q. Zeng R. Xie Z.. (2021). OsMADS23 phosphorylated by SAPK9 confers drought and salt tolerance by regulating ABA biosynthesis in rice. PloS Genet. 17, e1009699. doi: 10.1371/journal.pgen.1009699 34343171 PMC8363014

[B32] Livak K. J. Schmittgen T. D. (2001). Analysis of relative gene expression data using real-time quantitative PCR and the 2(-Delta Delta C(T)) method. Methods 25, 402–408. doi: 10.1006/meth.2001.1262 11846609

[B33] Love M. I. Wolfgang H. Simon A. (2014). Moderated estimation of fold change and dispersion for RNA-seq data with DESeq2. Genome Biol. 15, 550–571. doi: 10.1186/s13059-014-0550-8 25516281 PMC4302049

[B34] Luis R. J. Allyn K. B. Elliot M. M. (1996). Dimerization specificity of Arabidopsis MADS domain homeotic proteins APETALA1, APETALA3, PISTILLATA, and AGAMOUS. Proc. Natl. Acad. Sci. U. S. A. 93, 4793–4798. 8643482 10.1073/pnas.93.10.4793 PMC39358

[B35] Marie B. Claire H. Michel P. Wout B. (2003). Lignin: genetic engineering and impact on pulping. Crit. Rev. Biochem. Mol. Biol. 38, 305–350. doi: 10.1111/j.1365-313X.2008.03457.x 14551235

[B36] Mirzaghaderi G. (2024). Genome-wide analysis of MADS-box transcription factor gene family in wild emmer wheat (*Triticum turgidum subsp.* dicoccoides). PloS One 19, e0300159. doi: 10.1371/journal.pone.0300159 38451993 PMC10919676

[B37] Moura J. Bonine C. Viana J. Dornelas M. Mazzafera P. (2010). Abiotic and biotic stresses and changes in the lignin content and composition in plants. J. Integr. Plant Biol. 52, 360–376. doi: 10.3390/ijms19020335 20377698

[B38] Natalia C. Joel H. Wendy C. Maite A. Carlos T. Berenice G.. (2019). MADS-Box genes are key components of genetic regulatory networks involved in abiotic stress and plastic developmental responses in plants. Front. In Plant Sci. 10. doi: 10.3389/fpls.2019.00853 PMC6636334 31354752

[B39] Nie H. Wang Y. Wei C. Grover C. E. Su Y. Wendel J. F.. (2021). Embryogenic calli induction and salt stress response revealed by RNA-Seq in diploid wild species *Gossypium sturtianum* and *Gossypium raimondii* . Front. Plant Sci. 12. doi: 10.3389/fpls.2021.715041 PMC8424188 34512696

[B40] Okay A. Kırlıoğlu T. Durdu Y.Ş. Akdeniz S.Ş. Büyük İ. Aras E. S. (2024). Omics approaches to understand the MADS-box gene family in common bean (*Phaseolus vulgaris* L.) against drought stress. Protoplasma 4, 709–724. doi: 10.1007/s00709-024-01928-z PMC11196313 38240857

[B41] Pachamuthu K. Sundar V. Narjala A. Singh R. Das S. Pal H.. (2022). Nitrate-dependent regulation of miR444-OsMADS27 signaling cascade controls root development in rice. J. Exp. Bot. 73, 3511–3530. doi: 10.1093/jxb/erac083 35243491

[B42] Puig J. M. Meynard D. Khong G. N. Pauluzzi G. Guiderdoni E. Gantet P. (2013). Analysis of the expression of the *AGL17*-like clade of MADS-box transcription factors in rice. Gene Expression Patterns Gep. 13, 160–170. doi: 10.1016/j.gep.2013.02.004 23466806

[B43] Ryo T. Ayumi Y. Shino M. Miki N. Minami M. Masaaki S.. (2022). Brz-insensitive-pale green 1 is encoded by chlorophyll biosynthesis enzyme gene that functions in the downstream of brassinosteroid signaling. Biosci. Biotechnol. Biochem. 8, 1041–104. doi: 10.1093/bbb/zbac071 35583242

[B44] Simon A. Theodor P. P. Wolfgang H. (2015). HTSeq-a Python framework to work with high-throughput sequencing data. Bioinformatics 31, 166–169. doi: 10.1093/bioinformatics/btu638 25260700 PMC4287950

[B45] Su Y. Guo A. Huang Y. Wang Y. M. Hua J. P. (2020). *GhCIPK6a* increases salt tolerance in transgenic upland cotton by involving in ROS scavenging and MAPK signaling pathways. BMC Plant Biol. 20, 421–440. doi: 10.1186/s12870-020-02548-4 32928106 PMC7488661

[B46] Takahashi F. Mizoguchi T. Yoshida R. Ichimura K. Shinozaki K. (2011). Calmodulin-dependent activation of MAP kinase for ROS homeostasis in *Arabidopsis* . Mol. Cell 41, 649–660. doi: 10.1016/j.molcel.2011.02.029 21419340

[B47] Wang Q. Chen P. Wang H. Chao S. Guo W. Zhang Y.. (2023). Physiological and transcriptomic analysis of *OsLHCB3* knockdown lines in rice. Mol. Breed. 43, 38–53. doi: 10.1007/s11032-023-01387-z 37312752 PMC10248686

[B48] Wei X. Wang L. Yu J. Zhang Y. Li D. Zhang X. (2015). Genome-wide identification and analysis of the MADS-box gene family in sesame. Gene 569, 66–76. doi: 10.1016/j.gene.2015.05.018 25967387

[B49] Xu W. Dou Y. Geng H. Fu J. Dan Z. Liang T.. (2022). *OsGRP3* enhances drought resistance by altering phenylpropanoid biosynthesis pathway in rice (*Oryza sativa* L.). Int. J. Mol. Sci. 23, 7045–7061. doi: 10.3390/ijms23137045 35806050 PMC9266740

[B50] Yang Z. Nie G. Feng G. Xu X. Li D. Wang X.. (2022). Genome-wide identification of MADS-box gene family in orchardgrass and the positive role of *DgMADS114* and *DgMADS115* under different abiotic stress. Int. J. Biol. Macromol. 223, 129–142. doi: 10.1016/j.ijbiomac.2022.11.027 36356860

[B51] Yang G. Wang Y. Xia D. Gao C. Wang C. Yang C. (2014). Overexpression of a GST gene (*ThGSTZ1*) from *Tamarix hispida* improves drought and salinity tolerance by enhancing the ability to scavenge reactive oxygen species. Plant Cell Tiss Org. 117, 99–112. doi: 10.1007/s11240-014-0424-5

[B52] Yu W. Ji R. Feng R. Zhao X. Zhang Y. (2015). Response of water stress on photosynthetic characteristics and water use efficiency of maize leaves in different growth stage. Acta Ecologica Sinica 35, 2902–2909. doi: 10.5846/stxb201306101632

[B53] Yu N. Liang Y. Wang Q. Peng X. He Z. Hou X. (2022). Transcriptomic analysis of *OsRUS1* overexpression rice lines with rapid and dynamic leaf rolling morphology. Sci. Rep-UK. 12, 6736–6752. doi: 10.1038/s41598-022-10784-x PMC9038715 35468979

[B54] Zhang X. Fan B. Yu Z. Nie L. Zhao Y. Yu X.. (2019b). Functional analysis of three miRNAs in *Agropyron mongolicum* Keng under drought stress. Agronomy 9, 661–686. doi: 10.3390/agronomy9100661

[B55] Zhang D. Jiang S. Pan J. Kong X. Zhou Y. Liu Y.. (2014). The overexpression of a maize mitogen-activated protein kinase gene (ZmMPK5) confers salt stress tolerance and induces defence responses in tobacco. Plant Biol. 16, 558–570. doi: 10.1111/plb.12084 23952812

[B56] Zhang J. Wang S. Song S. Xu F. Pan Y. Wang H. (2019a). Transcriptomic and proteomic analyses reveal new insight into chlorophyll synthesis and chloroplast structure of maize leaves under zinc deficiency stress. J. Proteomics 199, 123–134. doi: 10.1016/j.jprot.2019.03.001 30849524

[B57] Zhao Y. Yun J. Shi F. Wang J. Yang Q. Chao Y. (2010). Molecular cloning and characterization of a group 3 LEA gene from *Agropyron mongolicum* Keng. Afr. J. Biotechnol. 9, 69–82. doi: 10.1186/1475-2859-9-69

