# Supplementary material for: AmMADS47 of Agropyron mongolicum negatively regulates drought tolerance in rice
Source: Front Plant Sci. 2025 May 1;16:1514134. doi: 10.3389/fpls.2025.1514134 (PMC12078277; doi:10.3389/fpls.2025.1514134)
Supplement: Supplementary Figure 1 — Positive identification of AmMADS47 overexpression vector. [file DataSheet1.zip › Supplementary files/Table S8.docx]

**Table S8** Statistics of DEGs information in four groups

| Treatment vs. control | Number of DEGs | Number of transcription factors |
| --- | --- | --- |
| OE vs. WT | 6,074 | 108 |
| OED vs. WTD | 1,620 | 21 |
| WT vs. WTD | 3,900 | 168 |
| OE vs. OED | 9,927 | 70 |
| Total | 21,521 | 367 |
