# Supplementary figures and images for: AmMADS47 of Agropyron mongolicum negatively regulates drought tolerance in rice
Source: Front Plant Sci. 2025 May 1;16:1514134. doi: 10.3389/fpls.2025.1514134 (PMC12078277; doi:10.3389/fpls.2025.1514134)

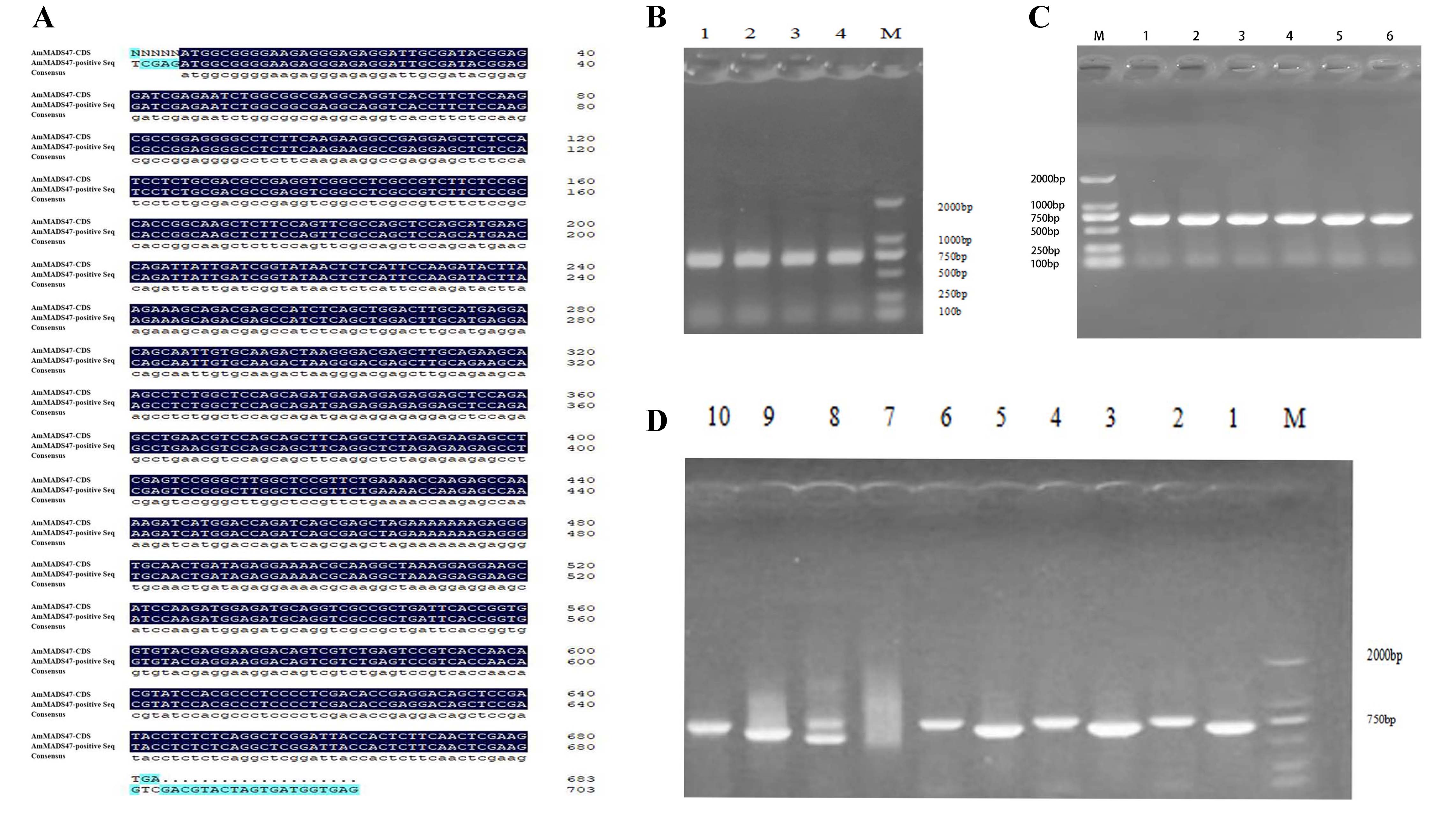

Supplement: Supplementary Figure 1 — Positive identification of AmMADS47 overexpression vector. [file DataSheet1.zip › Supplementary files/Figure S1.jpg]

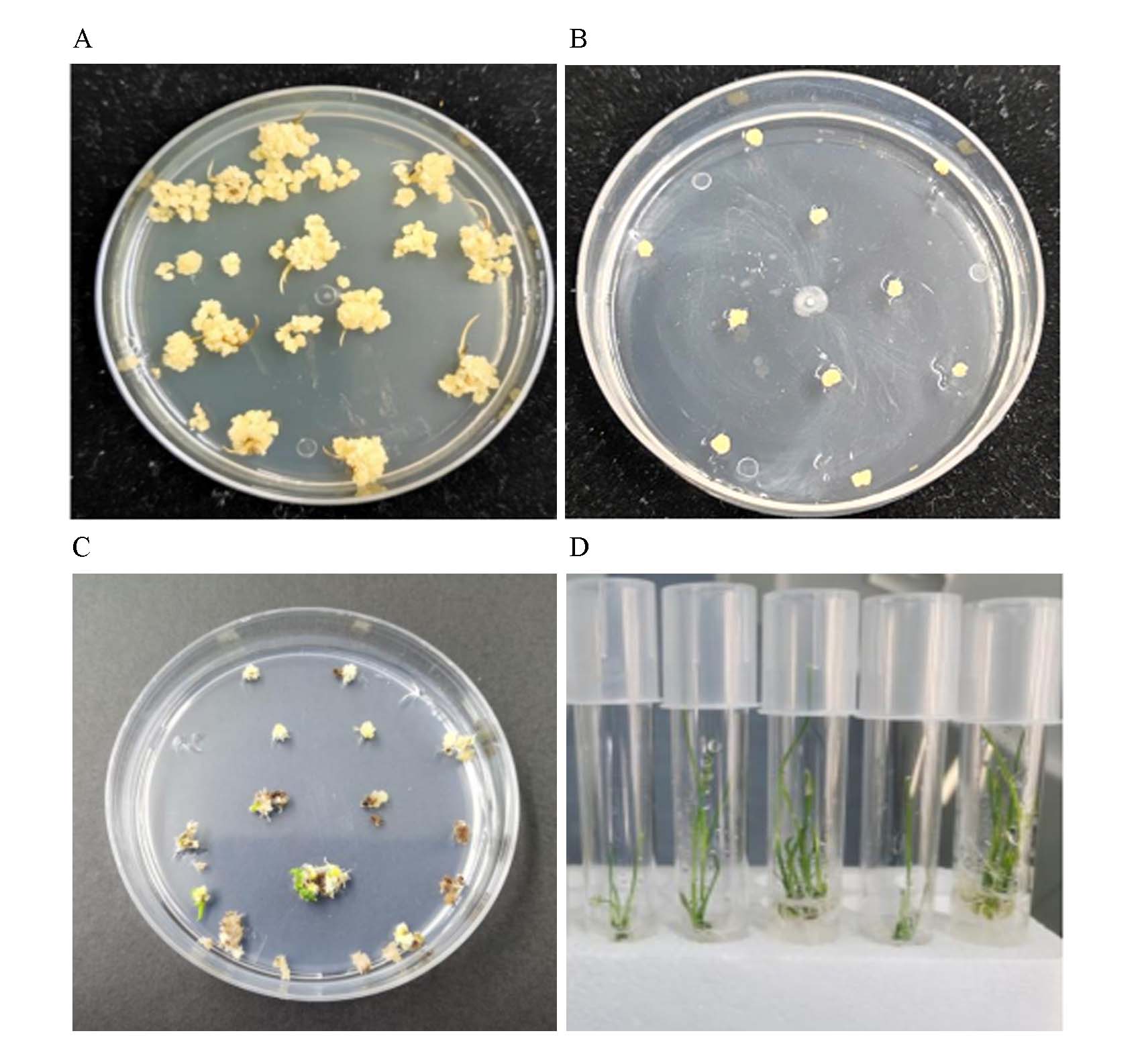

Supplement: Supplementary Figure 1 — Positive identification of AmMADS47 overexpression vector. [file DataSheet1.zip › Supplementary files/Figure S2.jpg]

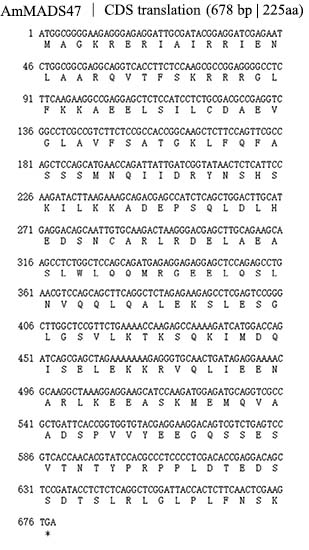

Supplement: Supplementary Figure 1 — Positive identification of AmMADS47 overexpression vector. [file DataSheet1.zip › Supplementary files/Figure S3.jpg]

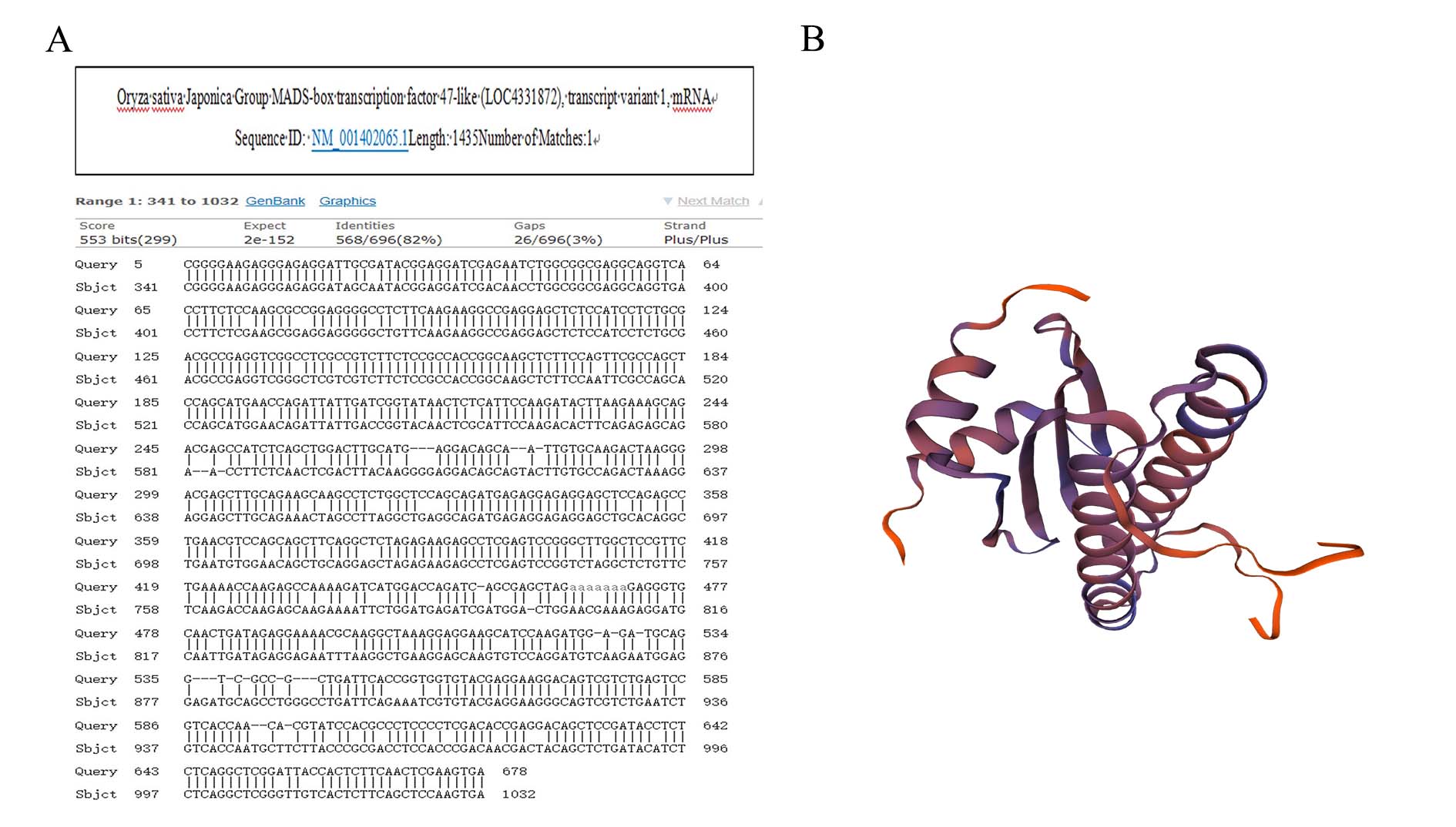

Supplement: Supplementary Figure 1 — Positive identification of AmMADS47 overexpression vector. [file DataSheet1.zip › Supplementary files/Figure S4.jpg]

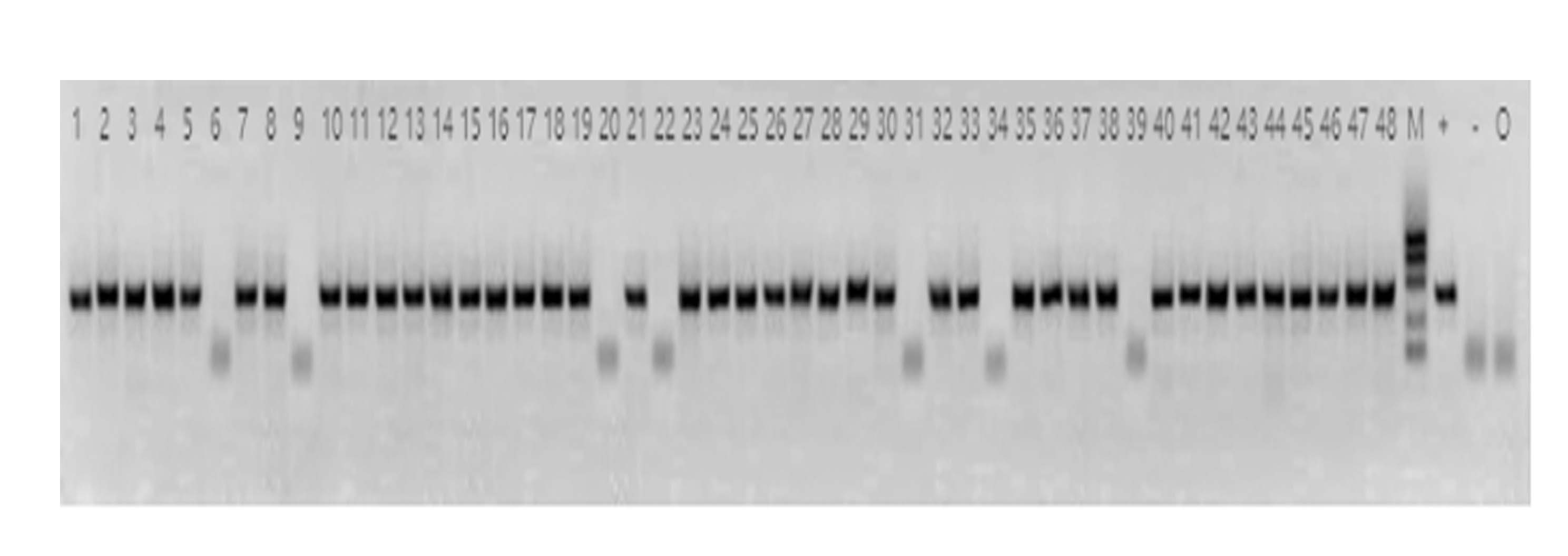

Supplement: Supplementary Figure 1 — Positive identification of AmMADS47 overexpression vector. [file DataSheet1.zip › Supplementary files/Figure S5.jpg]

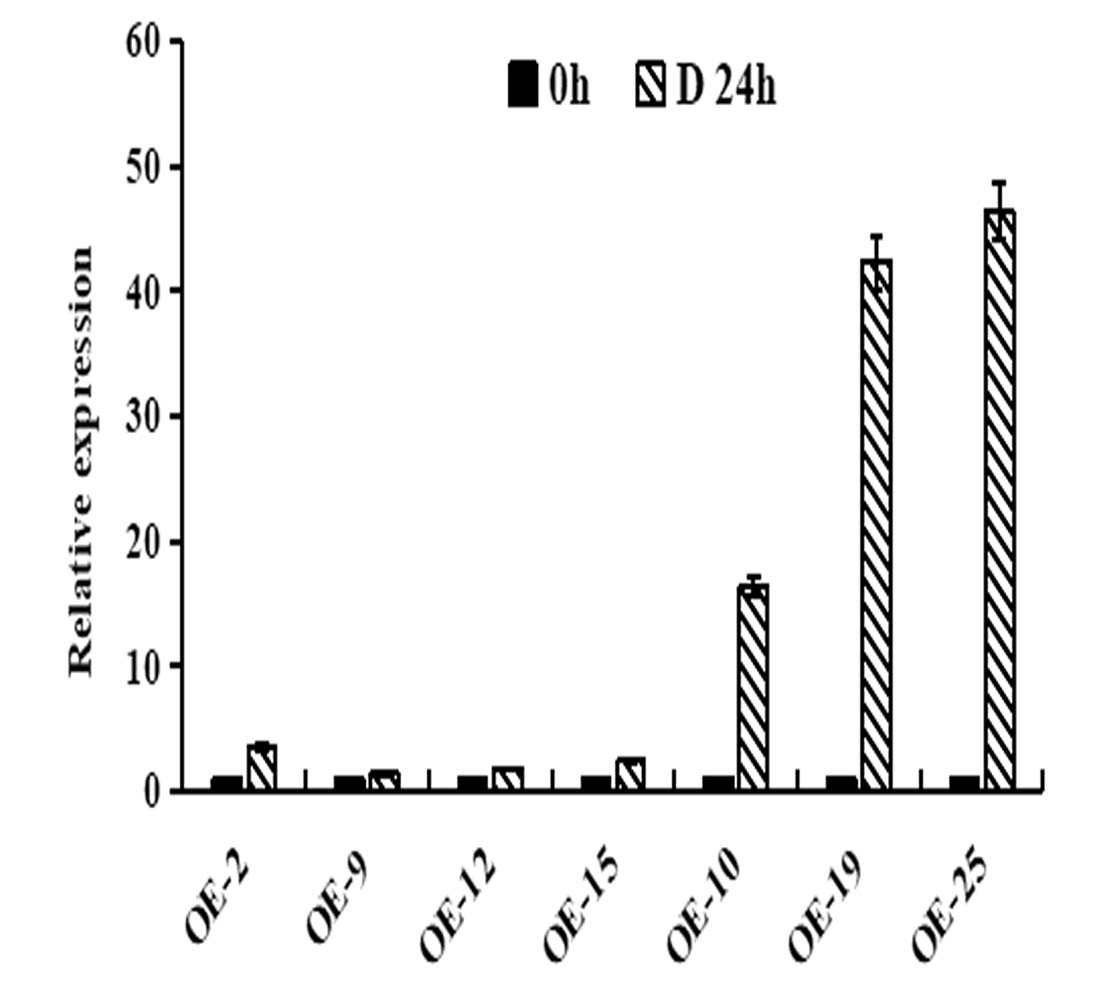

Supplement: Supplementary Figure 1 — Positive identification of AmMADS47 overexpression vector. [file DataSheet1.zip › Supplementary files/Figure S6.jpg]

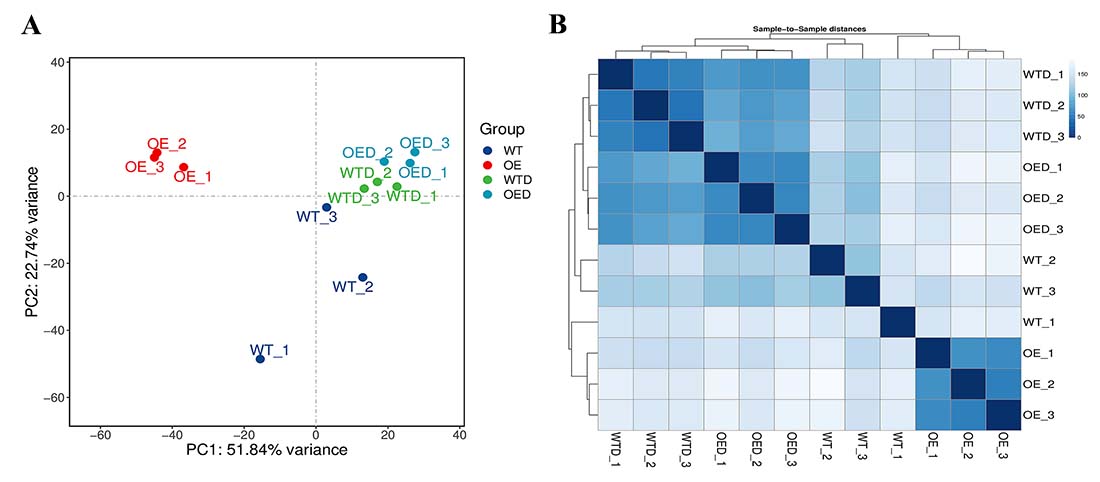

Supplement: Supplementary Figure 1 — Positive identification of AmMADS47 overexpression vector. [file DataSheet1.zip › Supplementary files/Figure S7.jpg]

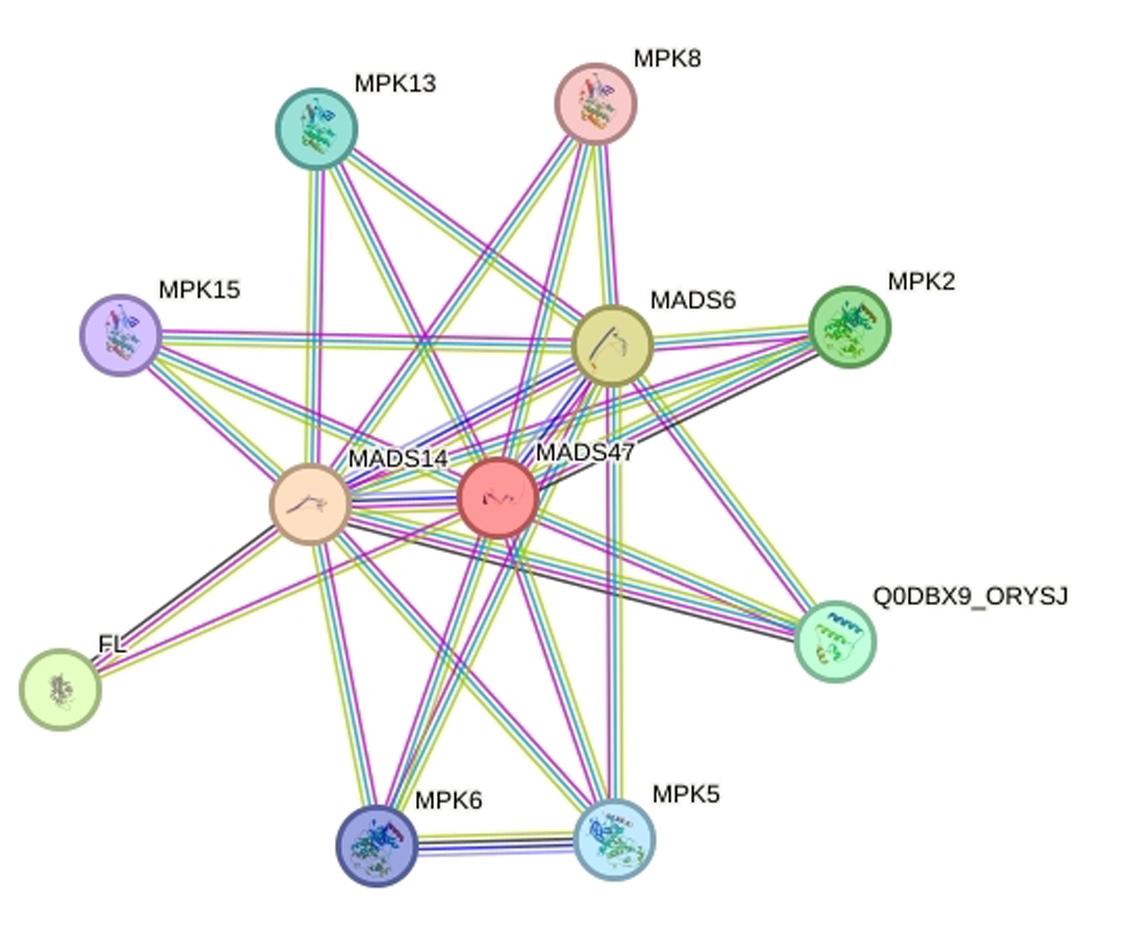

Supplement: Supplementary Figure 1 — Positive identification of AmMADS47 overexpression vector. [file DataSheet1.zip › Supplementary files/Figure S8.jpg]

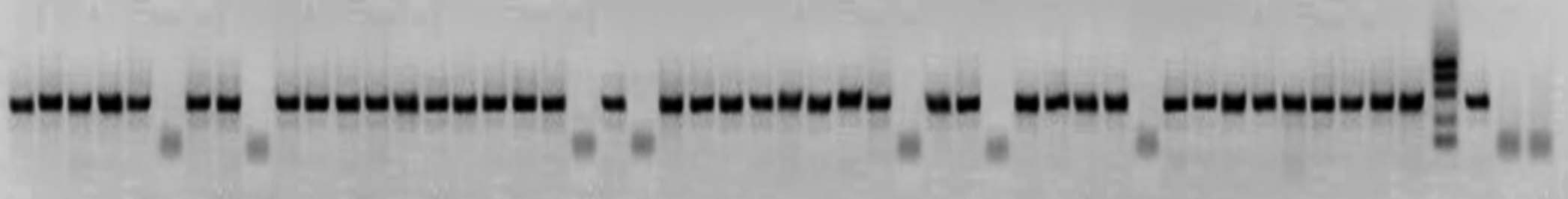

Supplement: Supplementary file 2 [file DataSheet2.zip › Original image of Figue S1 and S5/Original image of rice positive identification.jpg]

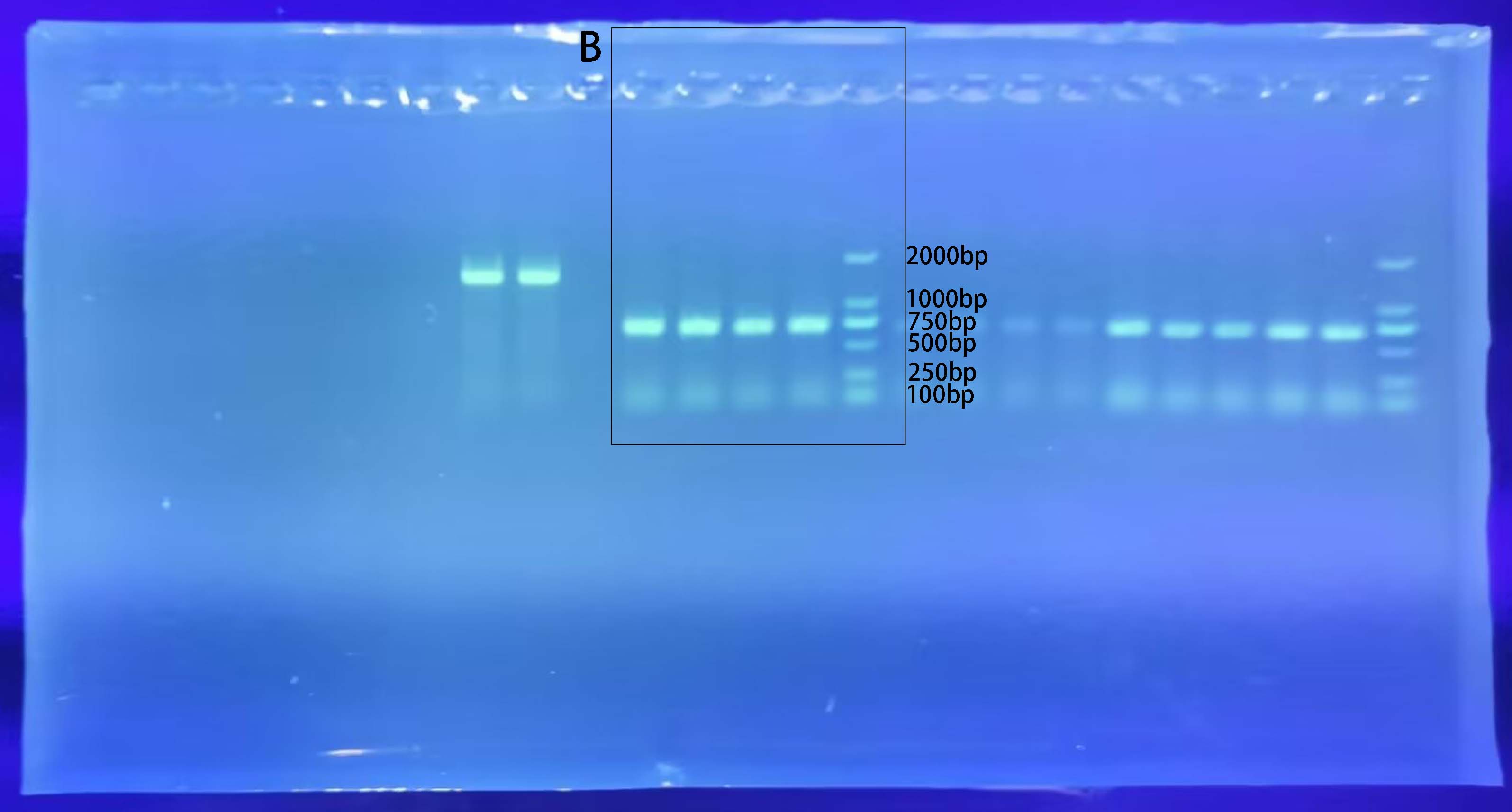

Supplement: Supplementary file 2 [file DataSheet2.zip › Original image of Figue S1 and S5/Supplementary Figure 1B.jpg]

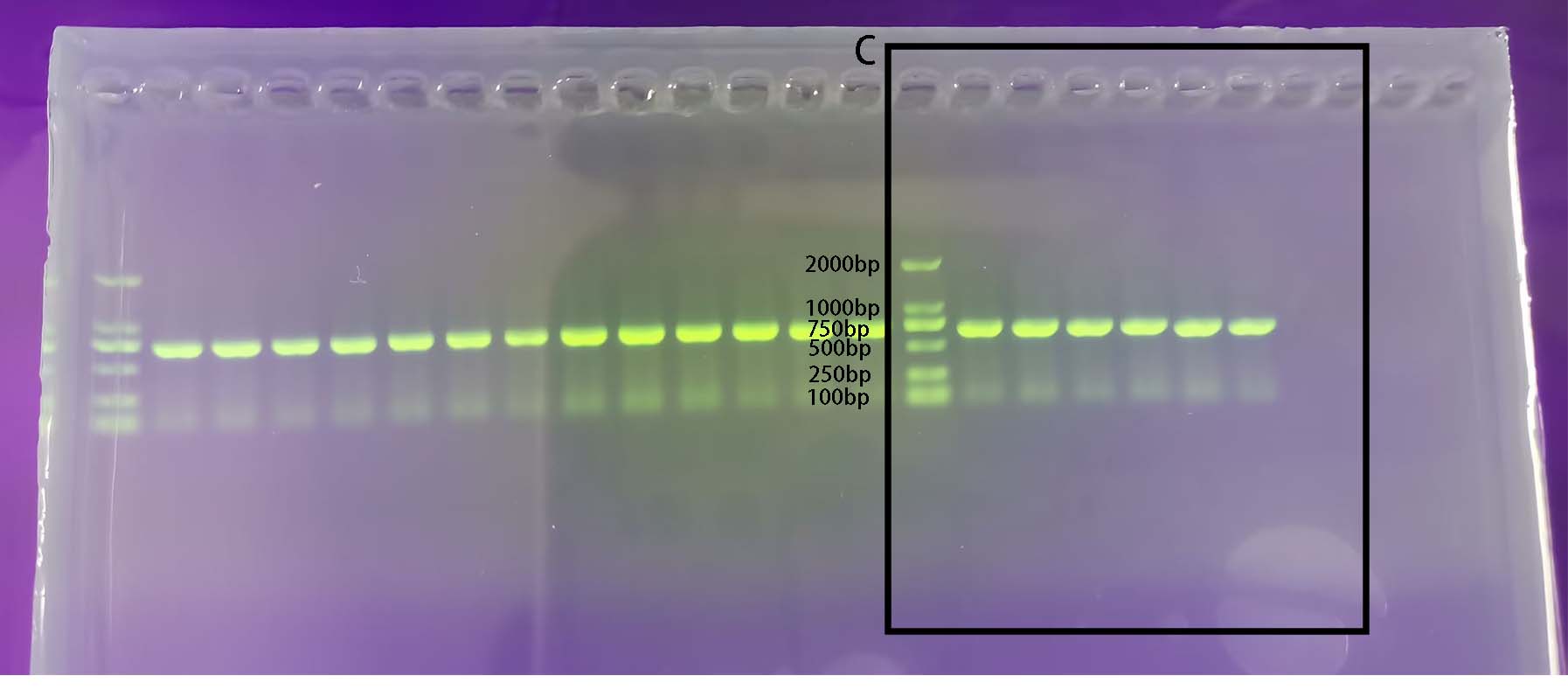

Supplement: Supplementary file 2 [file DataSheet2.zip › Original image of Figue S1 and S5/Supplementary Figure 1C.jpg]

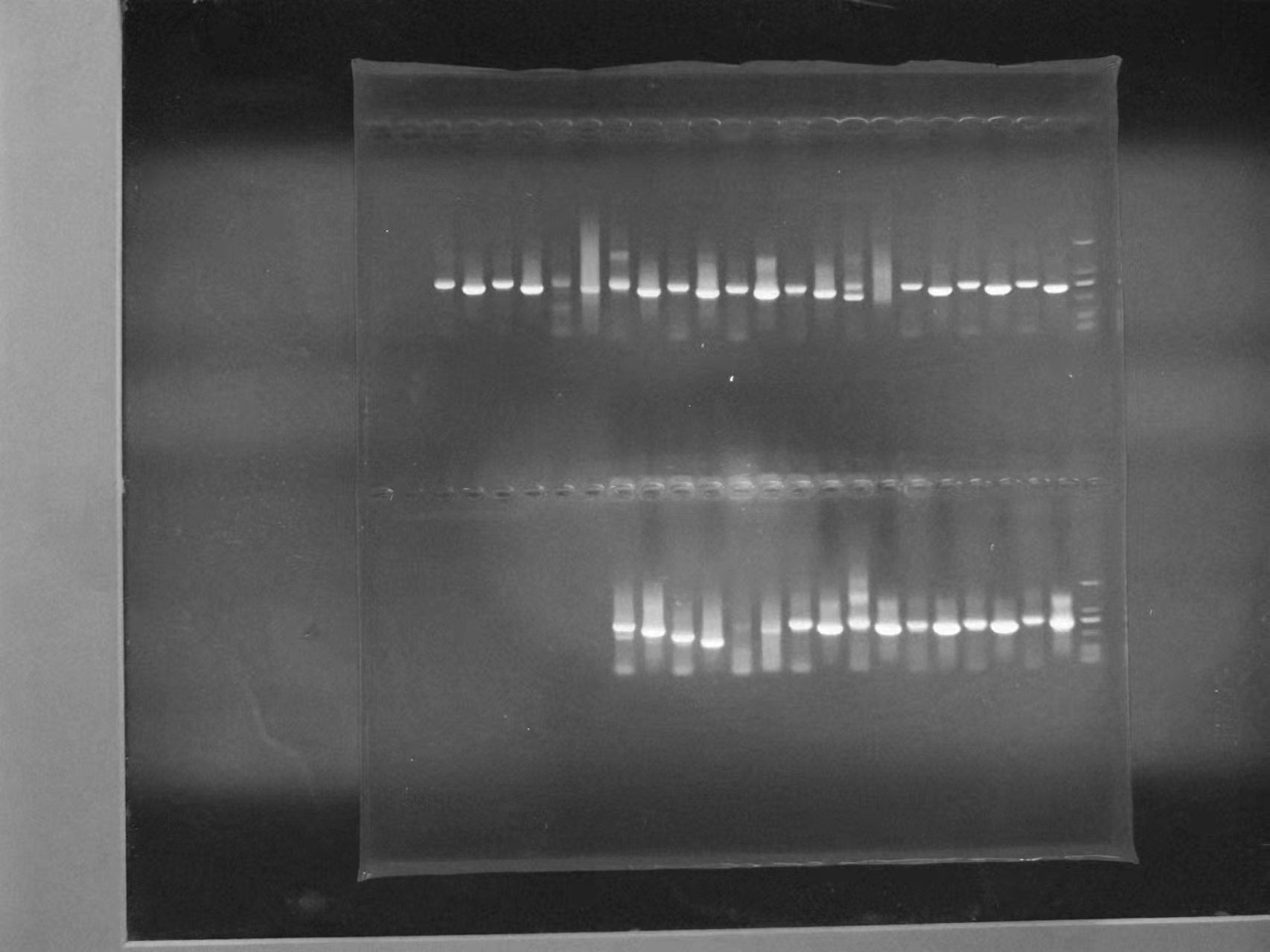

Supplement: Supplementary file 2 [file DataSheet2.zip › Original image of Figue S1 and S5/Supplementary Figure 1D.jpg]
